# Cepharanthine sensitizes human triple negative breast cancer cells to chemotherapeutic agent epirubicin via inducing cofilin oxidation-mediated mitochondrial fission and apoptosis

**DOI:** 10.1038/s41401-021-00715-3

**Published:** 2021-07-22

**Authors:** Li-wen Shen, Xiu-xing Jiang, Zhi-qiang Li, Jie Li, Mei Wang, Guan-fei Jia, Xin Ding, Ling Lei, Qi-hai Gong, Ning Gao

**Affiliations:** 1 Key Laboratory of Basic Pharmacology of Ministry of Education, Joint International Research Laboratory of Ethnomedicine of Ministry of Education, Zunyi Medical University, Zunyi, 563006 China; 2 College of Pharmacy, Army Medical University, Chongqing, 400038 China

**Keywords:** triple negative breast cancer, cepharanthine, epirubicin, mitochondrial superoxide, oxidative stress, cofilin, mitochondrial fission, apoptosis

## Abstract

Inhibition of autophagy has been accepted as a promising therapeutic strategy in cancer, but its clinical application is hindered by lack of effective and specific autophagy inhibitors. We previously identified cepharanthine (CEP) as a novel autophagy inhibitor, which inhibited autophagy/mitophagy through blockage of autophagosome-lysosome fusion in human breast cancer cells. In this study we investigated whether and how inhibition of autophagy/mitophagy by cepharanthine affected the efficacy of chemotherapeutic agent epirubicin in triple negative breast cancer (TNBC) cells in vitro and in vivo. In human breast cancer MDA-MB-231 and BT549 cells, application of CEP (2 μM) greatly enhanced cepharanthine-induced inhibition on cell viability and colony formation. CEP interacted with epirubicin synergistically to induce apoptosis in TNBC cells via the mitochondrial pathway. We demonstrated that co-administration of CEP and epirubicin induced mitochondrial fission in MDA-MB-231 cells, and the production of mitochondrial superoxide was correlated with mitochondrial fission and apoptosis induced by the combination. Moreover, we revealed that co-administration of CEP and epirubicin markedly increased the generation of mitochondrial superoxide, resulting in oxidation of the actin-remodeling protein cofilin, which promoted formation of an intramolecular disulfide bridge between Cys39 and Cys80 as well as Ser3 dephosphorylation, leading to mitochondria translocation of cofilin, thus causing mitochondrial fission and apoptosis. Finally, in mice bearing MDA-MB-231 cell xenografts, co-administration of CEP (12 mg/kg, ip, once every other day for 36 days) greatly enhanced the therapeutic efficacy of epirubicin (2 mg/kg) as compared with administration of either drug alone. Taken together, our results implicate that a combination of cepharanthine with chemotherapeutic agents could represent a novel therapeutic strategy for the treatment of breast cancer.

## Introduction

Oxidative stress, induced by reactive oxygen species (ROS), plays a crucial role in modulating a variety of cellular processes including cell proliferation, differentiation, apoptosis, etc [1–3]. ROS are molecules or ions that are formed by the incomplete one-electron reduction of oxygen, mainly including superoxide radical (O_2_^•-^), hydrogen peroxide (H_2_O_2_), and hydroxyl radical (^•^OH) [4]. Oxygen is initially converted to superoxide anion (O_2_^•-^), one of ROS, by electrons mainly leaked from complex II or complex III in the electron transport system present in mitochondria, where it is the major endogenous source of ROS [5]. ROS generated from mitochondria are released to the cytosol and results in damage to the cellular components including mitochondria and therefore leads to apoptosis [6]. These studies provide strong evidence that oxidative stress from mitochondria plays a critical role in induction of apoptosis, which is harmful in cancer settings. Cell signaling pathways regulated by ROS include effects on thiol groups and disulfide linkages to post-translationally modify protein structure to activate/inactivate specific kinase/phosphatase pathways [7, 8]. The mechanism for post-translational protein modification by ROS is a conformational change in the structure of protein kinases by formation of intramolecular disulfide bridges in cysteine (Cys) linkages. The oxidants such as taurine chloramine (TnCl) and H_2_O_2_ can cause damage to proteins with primary targets being Cys residues of proteins, resulting in mitochondrial damage and cell death [9]. The mechanism by which mitochondrially generated ROS (e.g., O_2_^•-^ and H_2_O_2_) modify the structure and function of these signaling proteins and exert pressure on cell death is not well understood.

Cofilin is an actin depolymerizing factor that regulates actin dynamics by increasing the rate of actin depolymerization and facilitating actin filament turnover [10, 11]. Cofilin activity is induced through its dephosphorylation at Ser3. Vice versa, phosphorylation of cofilin catalyzed by LIM-kinases inhibits its activity [12–14]. Only dephosphorylated cofilin (Ser3) can translocate from the cytosol to the mitochondria, leading to induction of apoptosis [15]. More recently, cofilin was found to be a key target for oxidation. Oxidants such as TnCl and H_2_O_2_ can induce oxidation of cofilin, resulting in formation of an intramolecular disulfide bridge between Cys39 and Cys80 of cofilin and its dephosphorylation at Ser3, leading, in turn, to mitochondrial damage and apoptosis [9]. Furthermore, mutation of Cys39 or Cys80 to glycine functionally mimic oxidation of cofilin, whereas mutation of Cys39 or Cys80 to alanine interferes with the oxidation of cofilin. A recent report showed that mitochondrial superoxide (O_2_^•-^) can induce formation of intra-molecular disulfide bridges, leading to oxidation of targeted proteins. The formation of disulfide bond was subsequently inhibited by superoxide dismutase [16, 17]. Thus, mitochondrial superoxide participates in the oxidative modification of targeted proteins and could be implicated in cellular signaling pathways. However, the molecular mechanisms by which mitochondrial superoxide induces oxidation of cofilin and ultimately leads to cell death remain unclear. Here we investigated whether mitochondrial superoxide influences the actin-remodeling activity of cofilin.

Autophagy is a catabolic process by which cells form double-membraned autophagic vesicles (AV) that sequester organelles and proteins and target them for degradation in the lysosome. Autophagy has dual roles in the regulation of cancer, acting as both a tumor suppressor by preventing the accumulation of damaged proteins and organelles, and as a mechanism of cell survival that can promote the growth of established tumors [18]. Increasing evidence reveals that the inhibition of autophagy enhances the efficacy of anticancer therapy, implying that autophagy inhibition is a potential valuable approach in combination with other anticancer therapeutic approaches to enhance cancer treatment [19]. Several phase I/II clinical studies involving autophagy inhibition using FDA-approved chloroquine or hydroxychloroquine in combination with chemotherapy for the treatment of different cancers, including breast cancer, are currently under way [20]. Cepharanthine, a benzylisoquinoline alkaloid extracted from *Stephania cepharantha* Hayata, is a natural anti-inflammatory and antineoplastic agent approved for clinically use to treat a variety of acute and chronic diseases, such as leukopenia, without major side effects [21]. In our previous studies, we identified cepharanthine as a novel autophagy inhibitor, which inhibited autophagy/mitophagy through blockage of autophagosome-lysosome fusion in human breast cancer cells [22]. Our findings suggest that inhibition of autophagy/mitophagy with cepharanthine potently enhances the efficacy of chemotherapy.

In the present study, we demonstrate that cepharanthine enhances the efficacy of chemotherapeutic agent epirubicin in its anti-tumor activities. Inhibition of autophagy/mitophagy by cepharanthine selectively enhances epirubicin-induced mitochondrial fission and apoptosis in triple negative breast cancer (TNBC) cells. Furthermore, cepharanthine increases sensitivity to epirubicin in mediating tumor regression in TNBC xenograft mouse model. Mechanistically, combination of cepharanthine/epirubicin induces mitochondrial superoxide species that represents a primary event resulting in oxidation of cofilin. In turn, this process leads to dephosphorylation and mitochondrial translocation of cofilin and culminates in mitochondrial fission and apoptosis. Our findings suggest that a combination of cepharanthine/epirubicin could represent a novel therapeutic strategy for treating TNBC.

## Materials and methods

### Cell culture, reagents and antibodies

MCF-10A, MDA-MB-231, MCF-7, and BT549 cells lines were obtained from the American Type Culture Collection (ATCC, Manassas, VA, USA). All cells were routinely cultured in Dulbecco’s modified Eagle’s medium (Gibco) or RPMI-1640 (Gibco) supplemented with 10% fetal bovine serum (FBS, Gibco) at 37 °C in a humidified atmosphere with 5% CO_2_.

Cepharanthine (A0653) was purchased from Must BioTechnology (Chengdu, China). Epirubicin (050-08981) was from Wako (Tokyo, Japan). Chloroquine diphosphate salt (C6628) was from Sigma-Aldrich (Gillingham, UK). Mitoquinone mesylate (HY-100116A) was purchased from Medchem Express (NJ, USA). Mn-TBAP (101386) was from Focus Biomolecules (PA, USA). Catalase (C3556) and sodium formate (V900189) were from Sigma-Aldrich (Gillingham, UK).

Primary antibodies used in this study were: Cleaved-Caspase 3 (9661), PARP (9532), phospho-Drp1 (4876), Drp1 (8570), ATG5 (12994), p62 (5114 S), phospho-Cofilin (3313), phospho-LIMK1/2 (3841), LIMK1 (3842), LIMK2 (3845), GAPDH (2118) were purchased from Cell Signaling Technology (Boston, MA, USA). VDAC1 (ab14734) was from Abcam (Cambridge, UK). Parkin (sc-32283), PINK1 (sc-33796), Cofilin (sc-376476), cytochrome *c* (sc-13156), Fis1 (sc-376466), Mff (sc-398617), Mfn1 (sc-166644), Mfn2 (sc-515647), and OPA1 (sc-393296) were purchased from Santa Cruz Biotechnology (Dallas, TX, USA). LC3 (L754S) was from Sigma-Aldrich (St. Louis, MI, USA); Secondary Goat-anti Rabbit (0741516) and Goat-anti Mouse (0741802) antibodies were purchased from Kirkegaard and Perry Laboratories (KPL, Gaithersburg, MD, USA).

### Cell viability assay

Cells were seeded in 96-well plates (3.0 × 10^3^/well). After treatment, 20 μL MTT (5 mg/mL) was added in each well and incubated at 37 °C for 4 h. After the medium was discarded, each well was supplemented with 150 μL DMSO to dissolve the formazan before being measured by a microplate reader at 490 nm. The cell viabilities were normalized to the control group.

### Soft agar assay

Sustainment gel was mixed with 0.6% agarose (Sigma-Aldrich) in a cell culture medium in 12-well plates. One-thousand cells were cultured in cultivate gel above concretionary sustainment gel (mixed with 0.3% agarose in cell culture medium with 10% FBS). After 28 days, the colonies were counted and photographed.

### Apoptosis assay

Apoptosis was examined by flow cytometry according to the manufacturer’s instructions (BD Biosciences PharMingen). Briefly, 1 × 10^6^ cells were washed twice with phosphate-buffered saline (PBS) and stained with 2 μL Annexin V-FITC (556419, BD) and 5 μL PI (P4170, Sigma) for 15 min at room temperature in the dark. Quantification of apoptotic cells was performed by flow cytometry using a FACScan cytofluorometer (BD Biosciences). Both early (Annexin V-positive, PI-negative) and late (Annexin V-positive and PI-positive) apoptotic cells were included in the cell death determinations.

### Transmission electronic microscopy

Cells were fixed in 2.5% glutaraldehyde at 4 °C overnight, washed three times with PBS, and then postfixed with 2% osmium tetroxide for 1.5 h at room temperature. After fixation, the samples were dehydrated through a series of ethanol concentrations and embedded in Epon 812 resin and then stained with uranyl acetate/lead citrate. The ultrastructure of cells was visualized under a transmission electron microscope (JEM-1400PLUS, Japan).

### Mitochondrial and cytosolic fractionation

Mitochondrial and cytosolic fractions were obtained as previously described [23]. Briefly, cell pellets were washed twice with PBS and resuspended in 5× Buffer A (20 mM HEPES, 10 mM KCl, 1.5 mM MgCl_2_, 1 mM EDTA, 1 mM EGTA, 1 mM Na_3_VO_4_). Cells were homogenized by passing them 15 times through a 22-gauge needle. The homogenate was centrifuged at 1000 × *g* at 4 °C for 10 min. The supernatant was then transferred and continued being centrifuged at 3500 × *g* at 4 °C for 10 min. The pellet fraction was considered the “mitochondrial” fraction. The supernatant fraction was then centrifuged at 120,000 × *g* at 4 °C for 10 min; the supernatant fraction was then considered the “cytosolic” fraction.

### Western blot analysis

The protein samples (15–30 μg) were separated using SDS-PAGE and transferred to PVDF membranes (Bio-Rad, 162–0177). After blocking with 5% fat-free dry milk in 1× Tris-buffered saline (TBS), the membrane was probed overnight with primary antibodies at 4 °C. Protein bands were detected by incubating with horseradish peroxidase-conjugated antibodies (Kirkegaard and Perry Laboratories, Gaithersburg, MD, USA).

### JC-1 assay

Cells were incubated with 500 μL JC-1 staining working solution for 30 min at 37 °C (C2005, Beyotime, Shanghai, China) according to the manufacturer’s instructions. The fluorescence labeled cells were washed using phosphate buffered saline (PBS) and the green and red fluorescence was observed by fluorescence microscope (Thermo Fisher Scientific, Waltham, MA, USA).

### Immunofluorescence

Cells were seeded on coverslips and cultured in 24-well plates for 24 h. After treatment for 48 h, mitochondria were stained with MitoTracker Deep Red FM (M22426, Molecular Probes, Carlsbad, USA) according to the manufacturer’s instructions. For mitochondrial translocation of cofilin, cells were stained with MitoTracker Deep Red FM for 30 min, after which cells were prepared for immunostaining by incubation with primary antibody against cofilin at 4 °C overnight. Cells were then incubated with secondary antibodies conjugated with Alexa Fluor 488 (A21206, Molecular Probes) for 1 h at 37 °C. Images were captured using a laser-scanning confocal microscope (Zeiss LSM 700, Jena, Germany). All images were analyzed by ImageJ software (MD, USA).

### Measurement of intracellular ROS and mitochondrial superoxide

To determine intracellular ROS production, cells were incubated with 10 μM CM-H_2_DCFDA (C6827, Molecular Probes) for 30 min, washed twice with cold PBS and detected by flow cytometry using a FACScan cytofluorometer (BD Biosciences). To determine mitochondrial superoxide production, cells were incubated with 2.5 μM MitoSOX (M36008, ThermoFisher Scientific) for 20 min, fluorescence was measured by flow cytometry at 488 nm_Ex_/585 nm_Em_. To determine mitochondrial superoxide by using immunofluorescence, cells were rinsed with PBS for three times and incubated with 5 μM MitoSOX™ Red (Molecular Probes, Life Technologies, Carlsbad, CA, USA) and 50 nM MitoTracker Green (M7514, Molecular Probes) for 30 min. The levels of mitochondrial superoxide were viewed by a confocal scanning microscopy (Zeiss LSM 700, Jena, Germany) at an excitation wavelength of 488 nm and an emission wavelength of 525 nm.

### RNA interference and site mutant

The target sequence of cofilin shRNA (5′-CCGGAAGGTGTTCAATGACATGAAACTCGAGTTTCATGTCATTGAACACCTTTTTTTG-3′) and ATG5-siRNA was constructed by Gene Chem Co. Ltd. (Shanghai, China). Cofilin-WT and site mutant plasmids of cofilin (Cofilin-S3A and Cofilin-S3E) were a gift from Prof James Bamburg (Colorado State University, USA). The mutant plasmids of cysteine to glycine: Cys39 to Gly39 (C39G-cofilin), Cys80 to Gly80 (C80G-cofilin) and cysteine to alanine: Cys39 to Ala39 (C39A-cofilin), Cys80 to Ala80 (C80A-cofilin) were constructed by Gene Chem Co. Ltd. (Shanghai, China). All constructs were verified by DNA-sequencing. All plasmids were co-transfected with pLP1, pLP2 and pLP/VSVG (Invitrogen) into 293FT cells using Lipofectamine 3000 (Invitrogen) according to the manufacturer’s instructions. The supernatant containing the lentivirus was harvested and infected with MDA-MB-231 and BT-549 cells. Cells were subsequently selected with 8 μg/mL puromycin to establish stable cell lines.

### Xenograft assay

Female nude mice (5–6 weeks old) were purchased from Vital River Laboratories (VRL, Beijing, China) and fed a standard animal diet and water. All animal experiments were carried out according to a protocol approved by the IACUC of Zunyi Medical University. MDA-MB-231 cells were suspended in a 1:1 ratio in DMEM medium with a Matrigel basement membrane matrix (Sigma, E1270). Cells (4 × 10^7^) were inoculated in the right legs of mice. After tumor inoculation, the mice were randomly divided into four treatment groups (16 mice per group; six mice were used for body weight and tumor volume measurement, the others for survival analysis). The mice were treated with either vehicle, cepharanthine (12 mg/kg) or epirubicin (2 mg/kg), or a combination of cepharanthine/epirubicin by intraperitoneal injection once every 2 days. The body weight and tumor volume (mm^3^) were measured. The mice were euthanized 36 days after medication, the tumors were excised and were either formalin-fixed or flash-frozen at −20 °C. The hematoxylin and eosin (H&E) staining, TUNEL and immunohistochemical analyses were performed as previously described [24]. For the H&E staining, the formalin-fixed tumors were embedded in paraffin blocks and visualized by optical microscope (DM4000B, Leica). For the TUNEL apoptosis staining, the fixed tumor sections were stained by the In Situ Cell Death Detection Kit (Roche Applied Science) according to the manufacturer’s protocol. For immunohistochemical analyses, fixed tumor sections were incubated with primary antibodies against Cleaved-Caspase-3, washed three times in PBS, incubated with biotinylated secondary antibody for 1 h, followed by incubation with a streptavidin-peroxidase complex for another 1 h. After three additional washes in PBS, diaminobenzidine working solution was applied. After counterstaining with DAPI, the expression of Cleaved-Caspase-3 in tumor sections was visualized by optical microscope (DM4000B, Leica).

### Statistical analysis

All data are representative of at least five separate experiments in vitro, and 16 mice (6 mice for body weight and tumor volume measurement, 10 mice for survival analysis) in vivo. Statistical differences were calculated by either Student’s *t*-test (two-tailed, unpaired) using GraphPad Prism 8.0 software. Survival curves were calculated using the Kaplan–Meier analysis and the difference between the survival curves was calculated by the log-rank test. *P* < 0.05, *P* < 0.01, and *P* < 0.001 were regarded as significant differences.

## Results

### Cepharanthine increases sensitivity to epirubicin in vitro

**Fig. 1:**
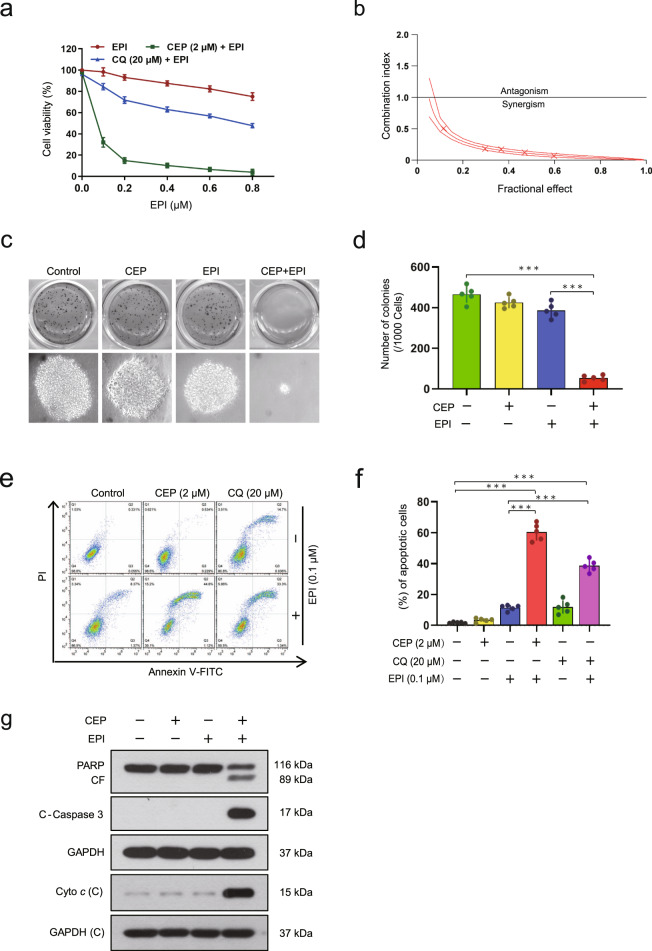
Cepharanthine increases sensitivity to epirubicin in vitro. **a** MDA-MB-231 cells were treated with various concentrations of epirubicin (EPI) in the presence or absence of 2 μM cepharanthine (CEP) or 20 μM CQ for 48 h, and MTT assays were performed to assess cell proliferation. **b** The combination index (CI) values for each fraction affected were determined using commercially-available software (Calcusyn, Biosoft). CI values less than 1.0 correspond to synergistic interactions. **c**, **d** Colony formation was detected using a soft agar assay in MDA-MB-231 cells treated without or with CEP (2 μM) or EPI (0.1 μM) or combination of CEP/EPI. **e**, **f** Cells were treated without or with 0.1 μM EPI in the presence or absence of 2 μM CEP or 20 μM CQ for 48 h, and Annexin V-FITC/PI staining and flow cytometry were employed to determine apoptosis. **g** Total cellular extract and cytosol fractions were prepared and subjected to Western blot using antibodies against total PARP, cleaved-PARP (CF), cleaved caspase 3 (C-Caspase 3), and cytochrome *c* (Cyto *c*). GAPDH was used as loading controls. Data represented as mean ± SD (*n* = 5, ***P < 0.001, Student’s two-tailed unpaired *t*-tests).

Growing evidence suggests that inhibiting autophagy may enhance the antitumor effect of chemotherapeutic agents [25]. Our previous study indicates that cepharanthine inhibits autophagy/mitophagy by blocking autophagosome-lysosome fusion in human breast cancer cells, suggesting that a combination of cepharanthine with classic chemotherapeutic agents (e.g., epirubicin) could represent a novel therapeutic strategy for the treatment of breast cancer [22]. We therefore investigated whether inhibiting autophagy through treatment with cepharanthine could sensitize breast cancer cells to chemotherapy. Cotreatment of MDA-MB-231 and BT549 cells with a nontoxic concentration of cepharanthine (2 μM) and minimal toxic concentrations of epirubicin led to significant decrease in cell viability compared with monotherapy alone (Fig. 1a and Supplementary Fig. S1a). In contrast, cotreatment with cepharanthine and epirubicin showed minimal decrease in cell viability of MCF-7 (estrogen-dependent) cells (Supplementary Fig. S1a) and normal breast epithelial MCF-10A cells (Supplementary Fig. S1b). The median dose effect analysis of cell viability in cells cotreated with cepharanthine and epirubicin for 48 h at fixed ratios yielded CI values consistently less than 1.0 in MDA-MB-231 and BT549 cells but greater than 1.0 in MCF-7 cells (Fig. 1b and Supplementary Fig. S1c). These results indicate that the combination of cepharanthine and epirubicin selectively inhibits cell proliferation in TNBC cells.

Chloroquine (CQ) or hydroxychloroquine (HCQ) is currently the only clinically available drug used to inhibit autophagy. Since cepharanthine has similar function to CQ, we compared the synergistic effects between cepharanthine/epirubicin and CQ/epirubicin on cell proliferation. Similar to cepharanthine, CQ has synergistic effect with epirubicin in inhibiting cell proliferation in TNBC cells (Fig. 1a and Supplementary Fig. S1a). Interestingly, we found that the synergistic effect of cepharanthine (2 μM) and epirubicin (0.1 μM) was more potent than that of CQ (20 μM) and epirubicin (0.1 μM), suggesting that a combination of cepharanthine with classical chemotherapeutic agents could represent a novel therapeutic strategy for treatment of TNBC.

Next, we determined the synergistic effect of cepharanthine and epirubicin on colony formation in both MDA-MB-231 and BT549 cells in vitro by using soft agar assay. Combination of cepharanthine/epirubicin significantly decreased colony formation compared with either control or monotherapy alone (Fig. 1c, d and Supplementary Fig. S2a, b).

To further evaluate the chemo-sensitizing characteristics of cepharanthine, we investigated the synergistic effect of cepharanthine and epirubicin on apoptosis in both TNBC cells by using Annexin V-FITC and propidium iodide (PI) double staining assays. Treating MDA-MB-231 and BT549 cells with cepharanthine or epirubicin alone minimally increased apoptosis (~5% to ~10%), whereas combination of cepharanthine/epirubicin markedly increased apoptosis (~50%) (Fig. 1e, f and Supplementary Fig. S2c, d). Similar results were also found in both TNBC cells treated with combination of CQ and epirubicin. However, the synergistic effect of cepharanthine (2 μM) and epirubicin (0.1 μM) was more potent than that of CQ (20 μM) and epirubicin (0.1 μM) (Fig. 1e, f and Supplementary Fig. S2c, d). Furthermore, Western blot revealed that combination of cepharanthine/epirubicin resulted in cleaved/activation of caspase 3, degradation of PARP, as well as release of cytochrome *c* from the mitochondria to the cytosol (Fig. 1g and Supplementary Fig. S2e). These results demonstrate that cepharanthine interacts synergistically with epirubicin to selectively induce apoptosis in TNBC cells via the mitochondrial pathway.

### Cepharanthine enhances the therapeutic efficacy of epirubicin in vivo

**Fig. 2:**
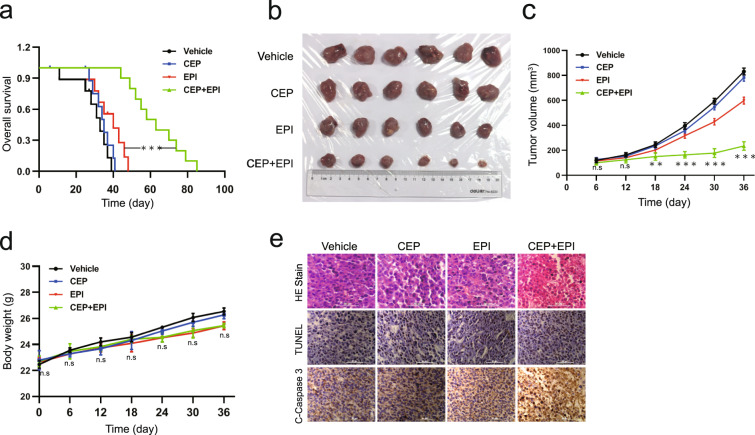
Cepharanthine enhances the therapeutic efficacy of epirubicin in vivo. Sixty-four BALB/c nude mice were inoculated subcutaneously with MDA-MB-231 cells and randomly divided into four groups (16 mice per group, 10 mice were used for determination of survival and 6 mice for determination of tumor volume and H&E, TUNEL, and immunohistochemistry analyses). **a** Comparison of the overall survival of mice between vehicle, cepharanthine, epirubicin and combination of cepharanthine/epirubicin (*n* = 10 mice per group). Statistical significance in survival was determined by log-rank test. ***P < 0.01, comparison between epirubicin and cepharanthine/epirubicin. **b**, **c** Tumor volumes were measured on the indicated days. Data represented as mean ± SD (*n* = 6, **P < 0.01, ***P < 0.001, ns not significant, Student’s two-tailed unpaired *t*-tests). **d** Body weight of mice was measure**d** on the indicated days. **e** Representative images of H&E, TUNEL, and immunohistochemistry staining for determination of morphology, apoptosis and expression of C-caspase 3 in xenograft tumor sections. Scar bar, 50 µm.

We used a mouse xenograft model to examine the synergistic effect of cepharanthine and epirubicin and determine whether our in vitro findings that cepharanthine can sensitize to epirubicin-induced cell death could be replicated in vivo. Once the implanted cells had developed into tumors after 6 days of inoculation, mice were received injections of either vehicle, cepharanthine (12 mg/kg), epirubicin (2 mg/kg) or both agents in combination for more than 80 days. Kaplan–Meir survival analysis revealed that the median survival time of the vehicle control group (*n* = 10) was approximately 33 days. Exposure of mice only to cepharanthine or epirubicin resulted in minor increases in their survival time (34.5 or 40 days, *n* = 10). However, a combination of cepharanthine/epirubicin significantly increased the median survival of the mice to 60.5 days (*P* < 0.001 compared to vehicle control or epirubicin treatment alone) (Fig. 2a). Single-agent treatment with either cepharanthine or epirubicin did not significantly reduced tumor volume relative to vehicle treatment (Fig. 2b, c). However, the combination of cepharanthine/epirubicin led to a significant reduction in tumor volume at day 12 relative to monotherapy alone (*P* < 0.05, Student’s *t* test, two-tailed, unpaired), and these events became more apparent at days 18, 24, 30 and 36 (*P* < 0.01 or *P* < 0.001) (Fig. 2b, c). No significant decrease in body weight or lethal toxicity was observed among vehicle control, cepharanthine or epirubicin treatment alone, and combination of cepharanthine/epirubicin (Fig. 2d).

To evaluate the synergistic effect of the combination on morphological change and apoptosis in MDA-MB-231 xenografts, hematoxylin and eosin (H&E) staining, TUNEL staining, and immunohistochemistry analyses were performed. H&E staining of sections of the tumors from mice treated with the cepharanthine/epirubicin combination showed significant changes in morphology, with signs of necrosis with infiltration of inflammatory cells and fibrosis (Fig. 2e, top panels). Either cepharanthine or epirubicin treatment alone caused a minimal increase in the number of TUNEL-positive cells (brown color) compared with vehicle control. However, combination of cepharanthine/epirubicin dramatically increased the number of TUNEL-positive cells compared with either vehicle control or single-agent treatment (Fig. 2e, middle panels). Furthermore, combination of cepharanthine/epirubicin markedly increased the immunoreactivity for cleaved caspase 3, indicative of apoptosis (Fig. 2e, bottom panels). Taken together, these data suggest that cepharanthine potently enhances the therapeutic efficiency of epirubicin in traditional TNBC therapy.

### Combination of cepharanthine/epirubicin impairs mitochondrial function

**Fig. 3:**
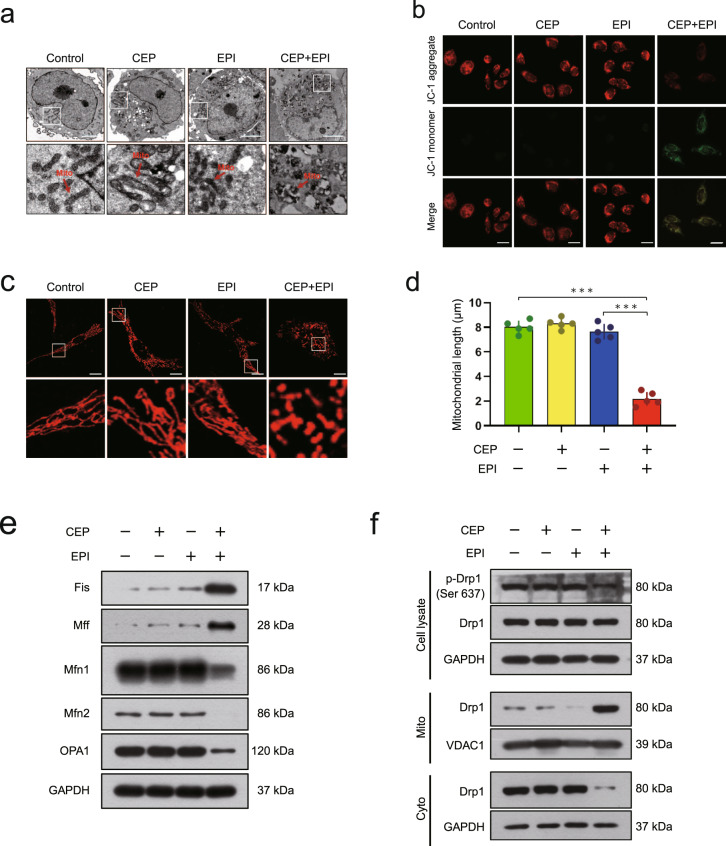
Combination of cepharanthine/epirubicin induces mitochondrial fission. MDA-MB-231 cells were treated without or with cepharanthine or epirubicin alone or combination of cepharanthine/epirubicin for 48 h. **a** Representative images of transmission electronic microscopy. **b** The mitochondrial membrane potential (MMP) was detected by JC-1 staining. **c** Mitochondrial morphology was determined by MitoTracker Red CMXRos staining and confocal microscopy. Scale bars, 10 μm. **d** Mitochondrial length was measured with ImageJ software. 50 cells of 5 independent experiments (mean ± SD, ***P < 0.001, Student’s two-tailed unpaired *t*-tests). **e** Western blot was performed to detect the expression of Fis1, MFF, Mfn1, Mfn2 and OPA1. GAPDH was used as loading control. **f** Whole cell lysates (Cell lysate), cytosolic (Cyto) and mitochondrial (Mito) fractions were prepared and subjected to Western blot by using antibody against Drp1 and phospho-Drp1. GAPDH and VDAC1 were used as loading control.

Increasing evidence indicates that abnormal mitochondrial function contributes to apoptosis induction [26, 27]. Our data indicated that combination of cepharanthine/epirubicin induced apoptosis which was accompanied by release of cytochrome *c*, suggesting that mitochondrial disfunction may contribute to apoptosis mediated by combination treatment. We therefore observed the synergistic effects of cepharanthine/epirubicin on the morphology of mitochondria by electron microscopy (EM). In control cells, the majority of mitochondria had normal structures with intact membranes and cristae. Cells treated with two-agents combination showed typical morphological changes of damaged swollen and fragmented mitochondria and distorted mitochondrial cristae (Fig. 3a and Supplementary Fig. S3a).

Loss of mitochondrial membrane potential (△ψm) is an important indicator of mitochondrial dysfunction [28]. We next examined the effects of cepharanthine/epirubicin combination on mitochondrial membrane potential through fluorescence microscopy by using JC-1 staining. Red fluorescence with high △ψm was observed in either control cells or cells treated with cepharanthine or epirubicin alone. Combination of cepharanthine/epirubicin significantly reduced △ψm as evidenced by a change in red to green fluorescence in TNBC cells (Fig. 3b and Supplementary Fig. S3b).

To further confirm whether combination of cepharanthine/epirubicin impairs mitochondrial function, we next evaluated the effects of these two-agents combination on mitochondrial dynamics using MitoTracker Red CMXRos. Treating MDA-MB-231 and BT549 cells with cepharanthine or epirubicin alone did not obviously affected the morphology of mitochondria. However, combination of cepharanthine/epirubicin significantly increased in the proportion of cells with fragmented mitochondria (*P* < 0.001 compared with either control or epirubicin treatment alone) (Fig. 3c, d and Supplementary Fig. S3c, d). In addition, combination of cepharanthine/epirubicin induced an upregulation of fission-related proteins Fis and Mff and downregulation of fusion-related proteins Mfn1, Mfn2 and OPA1 compared with control or single-agent treatment (Fig. 3e and Supplementary Fig. S3e).

Since dephosphorylation of Drp1 (Ser637) is involved in the regulation of mitochondrial fission through its mitochondrial translocation [29], we then examined the effects of cepharanthine/epirubicin combination on the phosphorylation of Drp1 (Ser637) and its mitochondrial translocation. As shown in Fig. 3f and Supplementary Fig. S3f, combined treating cells with cepharanthine/epirubicin decreased the levels of phospho-Drp1 (Ser637). Combination of cepharanthine/epirubicin also decreased the levels of Drp1 in the cytosol and increased the levels of Drp1 in the mitochondria. Together, our data indicate that combination of cepharanthine/epirubicin induces mitochondrial fission and apoptosis via dephosphorylation and mitochondrial translocation of Drp1.

### Excessive accumulation of mitophagosomes contributes to mitochondrial fission and apoptosis mediated by a combination of cepharanthine/epirubicin

**Fig. 4:**
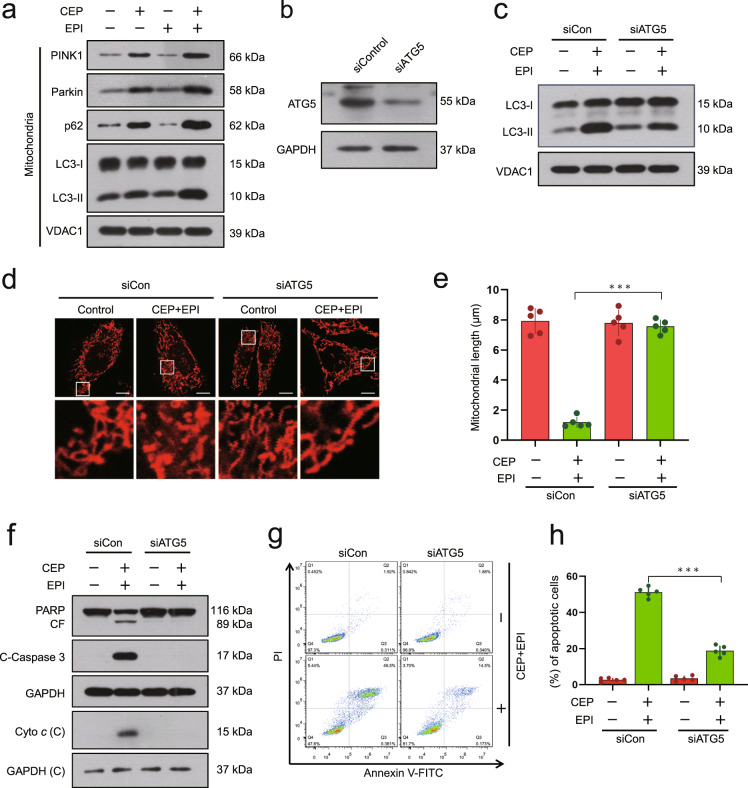
Excessive accumulation of mitophagosomes contributes to apoptosis induced by cepharanthine/epirubicin combination. **a** MDA-MB-231 cells were treated with cepharanthine in the presence or absence of epirubicin for 48 h, after which the mitochondrial fractions were prepared and subjected to Western blot analysis using antibodies against p62, LC3-I/LC3-II, PINK1 and Parkin. VDAC1 was used as loading control. **b** Cells were transfected with control siRNA (siControl) or siATG5, and Western blot analysis was used to determine the expression of ATG5. GAPDH was used as loading control. For **c–h**, cells stably expressing siControl or siATG5 were treated with cepharanthine in the presence or absence of epirubicin for 48 h. **c** The mitochondrial fractions were prepared and subjected to Western blot using antibodies against LC3-I/LC3-II. VDAC1 was used as loading control. **d** Mitochondrial morphology was determined by MitoTracker Red CMXRos staining and confocal microscopy. Scale bars, 10 μm. **e** Mitochondrial length was measured with ImageJ software. 50 cells of 5 independent experiments. **f** Western blot was performed to detect the expression of PARP, cleaved-PARP (CF), C-caspase 3 and cytochrome *c*. **g**, **h** Apoptosis was determined by Annexin V-FITC/PI staining and flow cytometry. Data represented as mean ± SD (*n* = 5, ***P < 0.001, Student’s two-tailed unpaired *t*-tests).

Our early data indicate that cepharanthine suppresses autophagic flux by blocking autophagosome-lysosome fusion [22]. We therefore investigated the effects of the combination of cepharanthine/epirubicin on the accumulation of mitophagosomes. Treatment of cells with cepharanthine alone increased the accumulation of LC3B-II and p62 in mitochondria, and combination of cepharanthine/epirubicin further enhanced LC3B-II and p62 accumulation in mitochondria (Fig. 4a and Supplementary Fig. S4a).

Since phosphatase and tensin homolog (PTEN)-induced kinase 1 (PINK1) and Parkin play crucial roles in the regulation of mitophagy [30], we next examined the effects of cepharanthine/epirubicin combination on accumulation of PINK1 and Parkin in the mitochondria. As shown in Fig. 4a and Supplementary Fig. S4a, treating cells with cepharanthine alone resulted in modest increases in levels of PINK1 and Parkin in the mitochondria. Combination of cepharanthine/epirubicin resulted in excessive accumulation of PINK1 and Parkin in mitochondria. These data suggest that PINK1/Parkin pathway is involved in the excessive accumulation of mitophagosomes mediated by combination of cepharanthine/epirubicin.

To further confirm the involvement of excessive accumulation of mitophagosomes in mitochondrial fission and apoptosis mediated by combination of cepharanthine/epirubicin, a siRNA approach was employed to stably knockdown ATG5 expression (Fig. 4b and Supplementary Fig. S4b). Knockdown of ATG5 markedly reduced combination-mediated LC3B-II accumulation in mitochondria (Fig. 4c and Supplementary Fig. S4c). Knockdown of ATG5 also abrogated combination-mediated mitochondrial fission (Fig. 4d, e and Supplementary Fig. S4d, e). Furthermore, knockdown of ATG5 abrogated combination-mediated degradation of PARP, cleavage/activation of caspase-3, cytochrome *c* release, as well as apoptosis (Fig. 4f–h and Supplementary Fig. S4f–h). Taken together, these findings indicate that the excessive accumulation of mitophagosomes is implicated in mitochondrial fission and apoptosis mediated by the combination of cepharanthine/epirubicin in TNBC cells.

### Combination of cepharanthine/epirubicin leads to dephosphorylation and mitochondrial translocation of cofilin

**Fig. 5:**
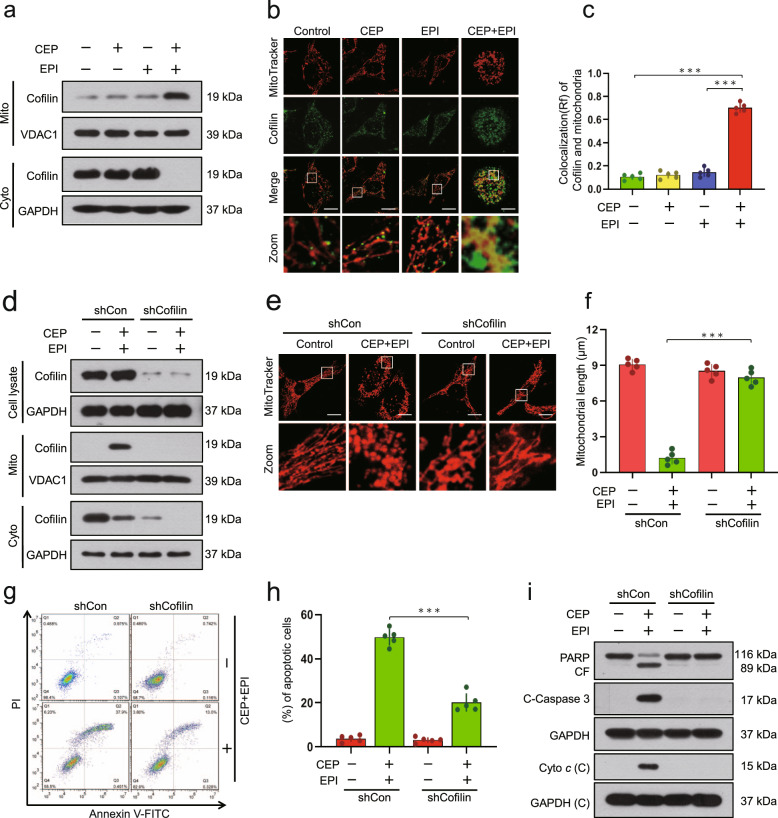
Combination of cepharanthine/epirubicin causes translocation of cofilin to the mitochondria. MDA-MB-231 cells were treated without or with cepharanthine or epirubicin alone or combination of cepharanthine/epirubicin for 48 h. **a** Cytosolic and mitochondrial fractions were prepared and subjected to Western blot using antibody against cofilin. GAPDH and VDAC1 were used as loading control. **b** Representative images of confocal microscopy which showed the colocalization of cofilin (green) and MitoTracker (red). Scale bars, 10 μm. **c** The Pearson’s correlation coefficient (*R*^2^) of cofilin and MitoTracker colocalization was from 50 cells of five independent experiments. For (**d–i**), MDA-MB-231 cells stably expressing Non-Target shRNA (shCon) or cofilin shRNA (shCofilin) were treated with or without combination of cepharanthine/epirubicin. **d** Whole cell lysates (Cell lysate), cytosolic (Cyto) and mitochondrial (Mito) fractions were prepared and subjected to Western blot by using antibody against cofilin. **e** Mitochondrial morphology was determined by MitoTracker Red CMXRos staining and confocal microscopy. Scale bars, 10 μm. **f** Mitochondrial length was measured with ImageJ software. 50 cells of 5 independent experiments. **g**, **h** Apoptosis was determined by Annexin V-FITC/PI staining and flow cytometry. **i** Western blot was performed to detect the expression of PARP, cleaved-PARP (CF), C-caspase 3 and cytochrome *c*. Data represented as mean ± SD (*n* = 5, ***P < 0.001, Student’s two-tailed unpaired *t*-tests).

Recent evidence reveals that mitochondrial translocation of cofilin results in mitochondrial fission and apoptosis [15]. Next, we determined the effects of cepharanthine/epirubicin combination on mitochondrial translocation of cofilin by Western blot. Cotreatment with cepharanthine and epirubicin resulted in increases in levels of cofilin in mitochondrial fractions and decreases in levels of cofilin in cytosolic fractions (Fig. 5a and Supplementary Fig. S5a). Immunofluorescence microscopy showed the cofilin signals in the mitochondria of cells treated with combination of cepharanthine/epirubicin (Fig. 5b, c and Supplementary Fig. S5b, c).

To further demonstrate the critical role that mitochondrial translocation of cofilin plays in the initiation of mitochondrial fission and apoptosis, we used shRNA to knock down cofilin protein levels in MDA-MB-231 and BT549 cells (Fig. 5d and Supplementary Fig. S5d). Knockdown of cofilin attenuated mitochondrial translocation of cofilin mediated by combination of cepharanthine/epirubicin (Fig. 5d and Supplementary Fig. S5d). Knockdown of cofilin also attenuated combination-mediated mitochondrial fission (Fig. 5e, f and Supplementary Fig. S5e, f). Furthermore, knockdown of cofilin significantly abrogated combination-mediated apoptosis, which was accompanied by PARP degradation, caspase 3 activation, as well as cytochrome *c* release (Fig. 5g–i and Supplementary Fig. S5g–i). Taken together, these findings suggest that mitochondrial translocation of cofilin is crucial for mitochondrial fission and apoptosis mediated by combination of cepharanthine/epirubicin.

**Fig. 6:**
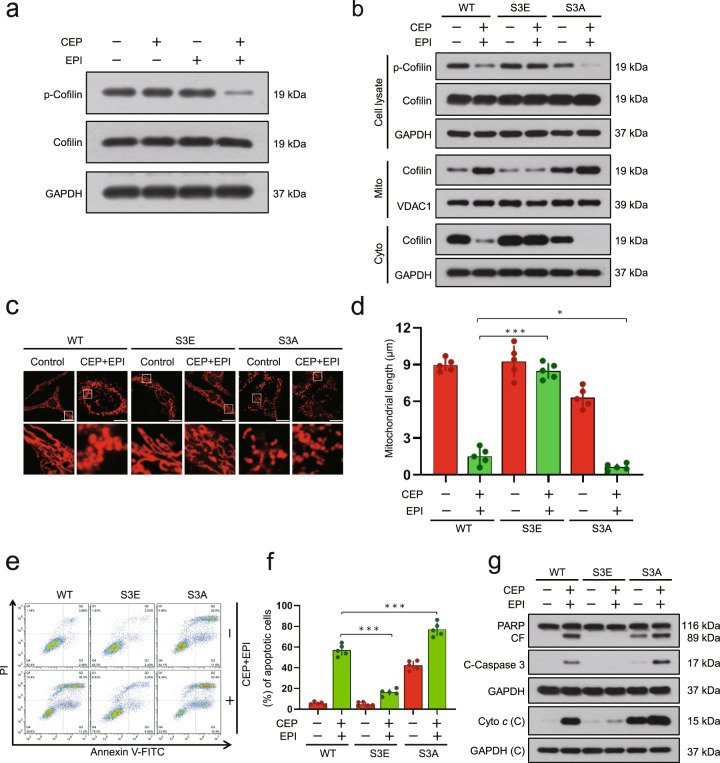
Dephosphorylation of cofilin contributes to combination-mediated mitochondrial fission and apoptosis. **a** MDA-MB-231 cells were treated without or with cepharanthine or epirubicin alone or combination of cepharanthine/epirubicin for 48 h, after which Western blot was employed to determine the levels of phospho-cofilin (p-Cofilin) and cofilin. For (**b**–**g**), MDA-MB-231 cells stably expressing Cofilin-WT or Cofilin-S3E or Cofilin-S3A were treated without or with combination of cepharanthine/epirubicin. **b** Whole cell lysates (Cell lysate), cytosolic (Cyto) and mitochondrial (Mito) fractions were prepared and subjected to Western blot by using antibody against cofilin and phospho-cofilin. **c** Mitochondrial morphology was determined by MitoTracker Red CMXRos staining and confocal microscopy. Scale bars, 10 μm. **d** Mitochondrial length was measured with ImageJ software. 50 cells of 5 independent experiments. **e**, **f** Apoptosis was determined by Annexin V-FITC/PI staining and flow cytometry. **g** Western blot was performed to detect the expression of C-PARP, C-caspase 3 and cytochrome *c*. Data represented as mean ± SD (*n* = 5, *P < 0.05, ***P < 0.001, Student’s two-tailed unpaired *t*-tests).

Increasing evidence suggests that only dephosphorylated cofilin is translocated to mitochondria, leading to the initiation of apoptosis [31, 32]. We therefore investigated the effect of cepharanthine/epirubicin combination on phosphorylation of cofilin. Exposure of MDA-MB-231 and BT549 cells to either cepharanthine or epirubicin alone did not affect the expression of phospho-cofilin (Ser3), whereas these two-agents combination led to decrease in levels of phospho-cofilin (Ser3) (Fig. 6a and Supplementary Fig. S6a). To further investigate whether the phosphorylation status of cofilin can influence its ability to translocate to mitochondria and induce apoptosis, two cofilin mutants were generated that mimic either the phosphorylated or dephosphorylated forms by changing Ser3 to aspartate (S3E) or alanine (S3A), respectively. The cofilin-S3E mutant, which mimics the phosphorylation form, reduced combination-mediated mitochondrial translocation of cofilin, mitochondrial fission, as well as apoptosis, whereas the cofilin-S3A mutant, which mimics dephosphorylation form, enhanced combination-mediated mitochondrial translocation of cofilin, mitochondrial fission, as well as apoptosis (Fig. 6b–g and Supplementary Fig. S6b–g). Together, our findings suggest that dephosphorylation of cofilin (Ser3) is crucial for combination-mediated mitochondrial translocation of cofilin, mitochondrial fission, as well as apoptosis.

### Combination of cepharanthine/epirubicin induces mitochondrial oxidative stress, leading to dephosphorylation of cofilin

**Fig. 7:**
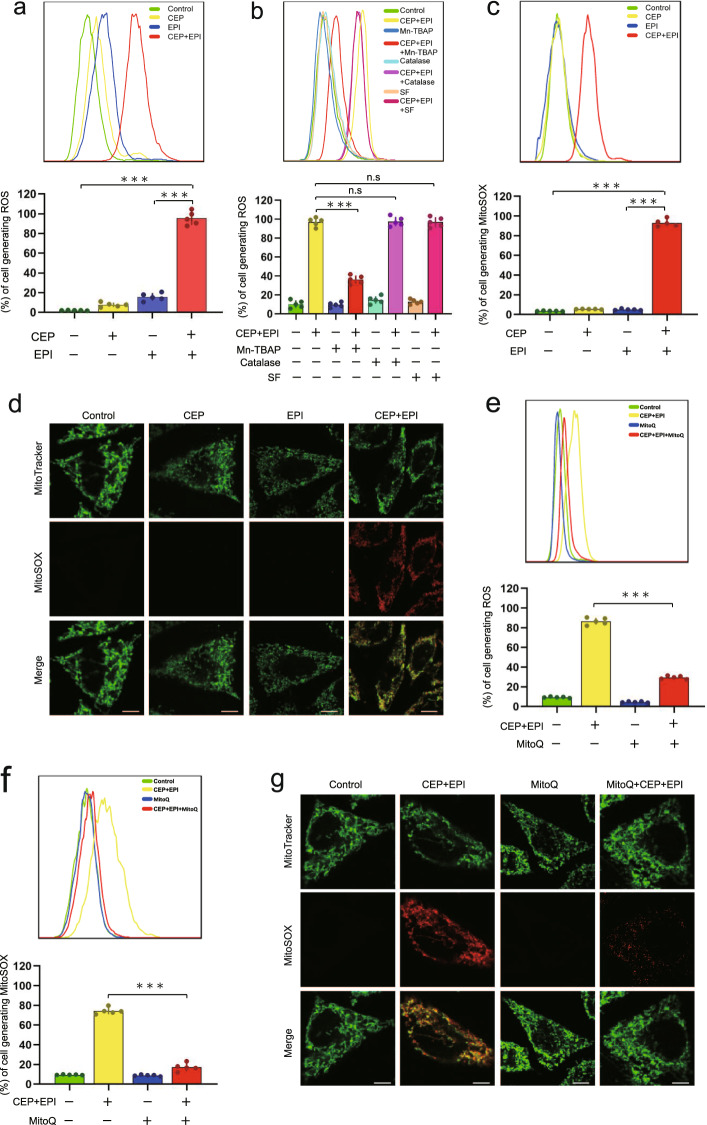
Combination of cepharanthine/epirubicin induces mitochondrial oxidative stress. **a** MDA-MB-231 cells were treated without or with cepharanthine or epirubicin alone or combination of cepharanthine/epirubicin for 48 h, after which ROS production was determined by DCFHDA staining and flow cytometry. **b** Cells were pretreated with antioxidants including as TBAP (200 μM), catalase (5000 U/mL), and sodium formate (SF, 2 mM) for 1 h, followed by treatment with combination of cepharanthine/epirubicin for 48 h, ROS production was determined by DCFHDA staining and flow cytometry. **c**, **d** Cells were treated in (**a**), mitochondrial superoxide production was determined by the fluorescent probes MitoSOX™ staining and flow cytometry. Scale bars, 10 μm. **e**, **f** Cells were pretreated with MitoQ (a mitochondrial targeted antioxidant), followed by treatment with combination of cepharanthine/epirubicin for 48 h, ROS and MitoSOX production was determined by DCFHDA or MitoSOX™ staining and flow cytometry. **g** The fluorescence intensity of mitochondrial superoxide as measured by MitoSOX and MitoTracker in cells treated without or with MitoQ or combination of cepharanthine/epirubicin. Scale bars, 10 μm. Data represented as mean ± SD (*n* = 5, ***P < 0.001, ns not significant, Student’s two-tailed unpaired *t*-tests).

Previous studies showed that oxidative stress leads to dephosphorylation of cofilin [9, 12]. We thus determined the effects of cepharanthine/epirubicin combination on the generation of reactive oxygen species (ROS) in MDA-MB-231 and BT549 cells. Flow cytometry analysis showed that treating cell with either cepharanthine or epirubicin alone caused a minimal increase in the generation of ROS. However, cepharanthine/epirubicin combination significantly increased the production of ROS compared with either control or single-agent treatment (Fig. 7a and Supplementary Fig. S7a). ROS, including superoxide radical (O_2_^•-^), H_2_O_2_, and hydroxyl radical (^•^OH), are recognized as signaling molecules that are mobilized in response to various stimuli [33]. To explore further the role of individual ROS on combination-mediated dephosphorylation of cofilin, we employed three antioxidants, for example, TBAP (a cell permeable SOD mimetic), catalase and sodium formate, which primarily act on O_2_^•-^, H_2_O_2_, and ^•^OH, respectively. Pretreatment with TBAP (an O_2_^•-^ scavenger) significantly abrogated combination-induced ROS generation. In contrast, catalase (a H_2_O_2_ scavenger) and sodium formate (an ^•^OH scavenger) failed to abrogate combination-mediated ROS generation in these cells (Fig. 7b and Supplementary Fig. S7b). Our data indicates that superoxide radical (O_2_^•-^) could be involved in combination-mediated dephosphorylation of cofilin.

Accumulating evidence suggests that mitochondria are the major producer of superoxide and other downstream ROS in the cell [34, 35]. We then examined whether mitochondria are the source of superoxide in combination-treated cells. Flow cytometry analysis showed that the levels of mitochondrial superoxide, as measured by MitoSOX, was significantly increased in cells treated with cepharanthine/epirubicin combination (Fig. 7c and Supplementary Fig. S7c). Immunofluorescence analysis also showed the colocalization of the MitoSOX Red fluorescence and MitoTracker Green signal in cells treated with combination of cepharanthine/epirubicin compared with that in either control or single-agents-treated cells (Fig. 7d and Supplementary Fig. S7d). Of note, pretreatment with mitoquinone (MitoQ, a mitochondrial targeted antioxidant) significantly attenuated the elevation of ROS in cells treated with combination (Fig. 7e and Supplementary Fig. S7e), suggesting that increased ROS are mainly derived from mitochondria. Similarly, pretreatment with MitoQ significantly attenuated the elevation of MitoSOX in cells treated with combination (Fig. 7f, g and Supplementary Fig. S7f, g). These results indicate that combination of cepharanthine/epirubicin could cause mitochondrial oxidative stress in TNBC cells.

**Fig. 8:**
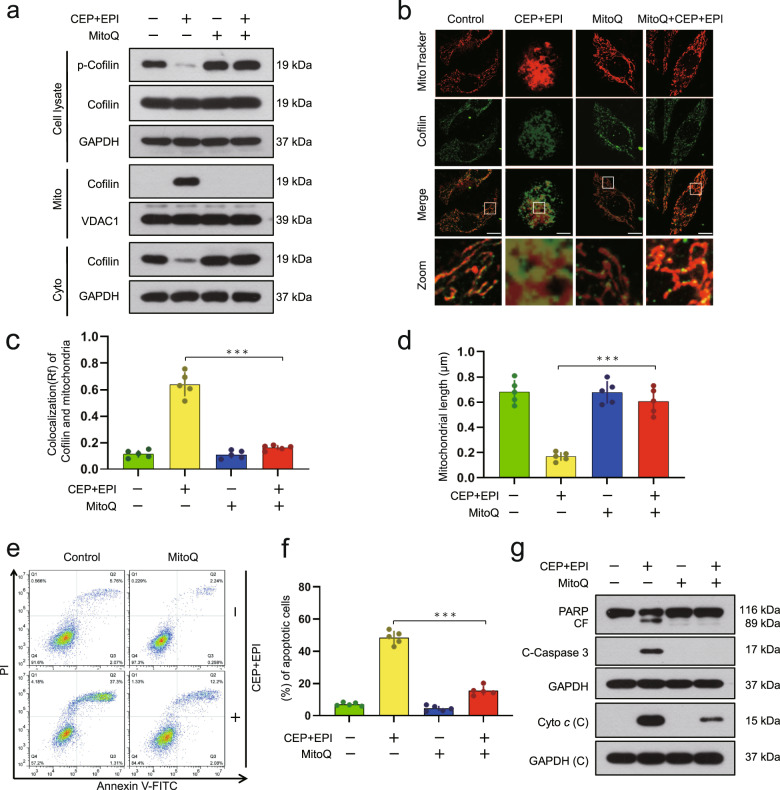
Mitochondrial superoxide leads to dephosphorylation and mitochondrial translocation of cofilin. MDA-MB-231 cells were treated without or with combination of cepharanthine/epirubicin in the presence or absence of MitoQ. **a** Whole cell lysates, cytosolic or mitochondrial fractions were prepared and subjected to Western blot using antibodies against phospho-cofilin (Ser3) and cofilin. **b** Representative images of confocal microscopy which showed the colocalization of cofilin (green) and MitoTracker (red). Scale bars, 10 μm. **c** The Pearson’s correlation coefficient (*R*^2^) of cofilin and MitoTracker colocalization was from 50 cells of five independent experiments. **d** Mitochondrial length was measured with ImageJ software. 50 cells of 5 independent experiments. **e**, **f** apoptosis was determined by Annexin V-FITC/PI staining and flow cytometry. **g** Western blot was performed to determine the expression of PARP, cleaved-PARP (CF), C-caspase 3 and cytochrome *c*. Data represented as mean ± SD (*n* = 5, ***P < 0.001, Student’s two-tailed unpaired *t*-tests).

To explore further the functional role of mitochondrial superoxide in combination-mediated dephosphorylation of cofilin and apoptosis in TNBC cells, MitoQ was employed. Pretreating MDA-MB-231 and BT549 cells with MitoQ markedly attenuated cepharanthine/epirubicin combination-mediated dephosphorylation of cofilin. MitoQ treatment also attenuated combination-mediated mitochondrial translocation of cofilin (Fig. 8a–c and Supplementary Fig. S8a–c). Furthermore, MitoQ treatment significantly attenuated the combination-induced mitochondrial fission (Fig. 8b, d and Supplementary Fig. S8b, d). Finally, MitoQ treatment significantly attenuated the combination-induced apoptosis, PARP degradation, caspase 3 activation, as well as cytochrome *c* release (Fig. 8e–g and Supplementary Fig. S8e–g). Together, these findings support the theory that cepharanthine/epirubicin combination-mediated mitochondrial oxidative stress represents a primary cause of dephosphorylation of cofilin, resulting in its mitochondrial translocation. In turn, this oxidative stress leads to mitochondrial fission and apoptosis.

### Cepharanthine/epirubicin combination-induced mitochondrial superoxide leads to oxidation of cofilin

**Fig. 9:**
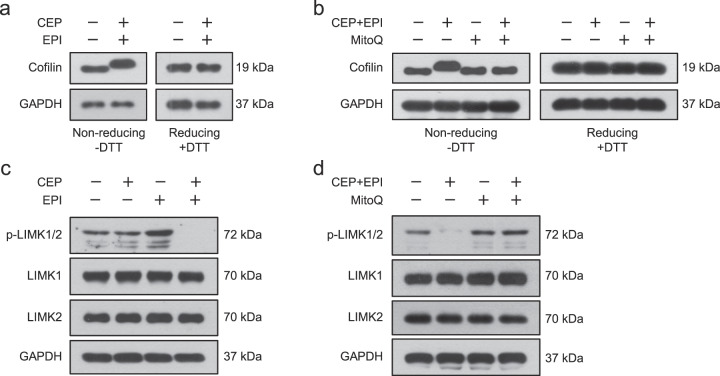
Combination-mediated mitochondrial superoxide leads to oxidation of cofilin and inactivation of LIM kinase. **a** MDA-MB-231 cells were treated without or with combination of cepharanthine/epirubicin, the expression of cofilin was analyzed either by nonreducing SDS-PAGE (left, DTT) or by reducing SDS-PAGE (right, +DTT). **b** Cells were treated without or with combination of cepharanthine/epirubicin in the presence or absence of MitoQ, the expression of cofilin was analyzed either by nonreducing SDS-PAGE (left, DTT) or by reducing SDS-PAGE (right, +DTT). **c** MDA-MB-231 cells were treated without or with cepharanthine or epirubicin alone or combination of cepharanthine/epirubicin for 48 h, after which Western blot was performed to determine the expression of phospho-LIMK1/2, LIMK1 and LIMK2. **d** Cells were treated without or with combination of cepharanthine/epirubicin in the presence or absence of MitoQ, the expression of phospho-LIMK1/2 was analyzed by Western blot.

Recent research reveals that cofilin itself is a target of oxidation, and cofilin is oxidized by oxidative stress, leading to loss of cofilin phosphorylation (Ser3) [12]. Our study showed that combination-induced mitochondrial superoxide contributes to dephosphorylation of cofilin, we speculate that the oxidation of cofilin may be crucial for the loss of its phosphorylation and subsequent translocation into the mitochondria. Formation of an intramolecular disulfide bridge usually leads to a conformational change of cofilin [12]. To analyze whether mitochondrial oxidative stress induced oxidation of cofilin, we performed Western blot analysis by adding with or without a disulfide bridge-breaking agent DTT. Without addition of DTT, the cell extract taken from combination-treated TNBC cells showed a higher-molecular weight species of cofilin compared with control cells (Fig. 9a, left and Supplementary Fig. S9a, left). This combination-induced conformational change of cofilin could be completely reversed by the addition of DTT to the sample before SDS-PAGE (Fig. 9a, right and Supplementary Fig. S9a, right). To further determine whether mitochondrial superoxide leads to oxidation of cofilin in cells-treated with combination, both TNBC cells were pretreated with MitoQ, followed by treatment with combination. After which, cell lysates were then analyzed by nonreducing SDS-PAGE. Pretreating cells with MitoQ abrogated combination-induced oxidation of cofilin, which showed a similar conformational change of cofilin as observed in control cells (Fig. 9b and Supplementary Fig. S9b). These results indicate that combination-mediated mitochondrial superoxide leads to oxidation of cofilin.

### Cepharanthine/epirubicin combination-mediated oxidation of cofilin prevents its phosphorylation (Ser3) by inactivation of LIM kinase

It has been shown that cofilin is inactivated by Ser3 phosphorylation by LIM motif-containing protein kinases (LIMKs) [36]. We next examined the synergistic effect of cepharanthine and epirubicin on phosphorylation of LIMK. Treating TNBC cells with either cepharanthine or epirubicin alone did not modify phosphorylation of LIMK, whereas combination treatment efficiently reduced phosphorylation of LIMK (Fig. 9c and Supplementary Fig. S9c). To further confirm whether combination-induced mitochondrial superoxide leads to inhibiting phosphorylation of LIMK, MitoQ was employed. Interestingly, pretreating cells with MitoQ effectively attenuated combination-inhibited phosphorylation of LIMK (Fig. 9d and Supplementary Fig. S9d). These results indicate that combination-inhibited phosphorylation of LIMK as a consequence of cofilin oxidation prevents its Ser3 phosphorylation.

### Cysteine 39 and Cysteine 80 are key sites for combination-mediated dephosphorylation and mitochondrial translocation of cofilin

**Fig. 10:**
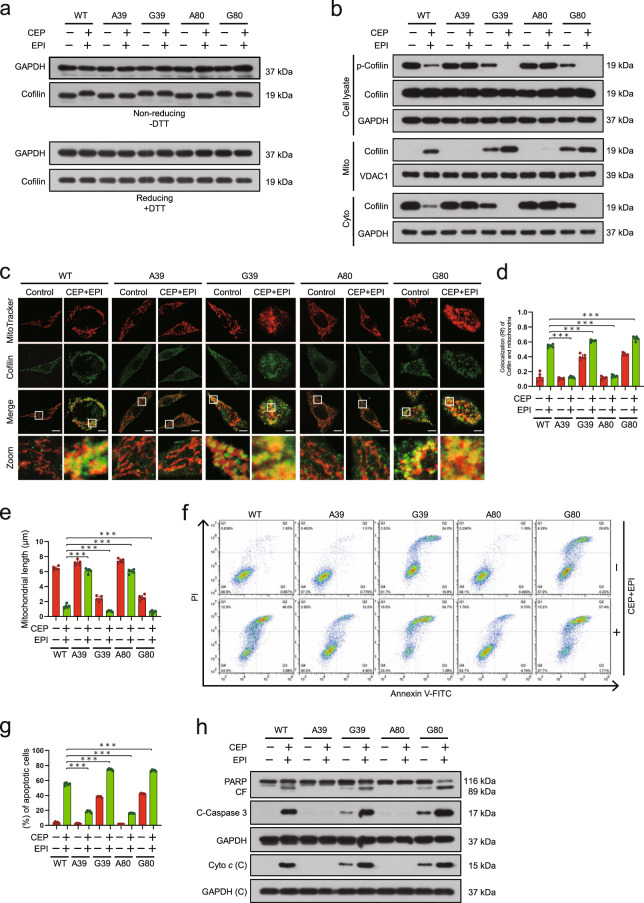
Cys39 and Cys80 are key sites for combination-mediated dephosphorylation and mitochondrial translocation of cofilin. MDA-MB-231 cells expressing either WT-cofilin or cysteine-to-glycine mutants of cofilin (C39G and C80G) or cysteine-to-Ala (C39A and C80A) were treated without or with combination of cepharanthine/epirubicin for 48 h. **a** The expression of cofilin was analyzed either by nonreducing SDS-PAGE (left, DTT) or by reducing SDS-PAGE (right, +DTT). **b** Whole cell lysates, cytosolic and mitochondrial fractions were prepared and subjected to Western blot by using antibodies against phospho-cofilin and cofilin. **c** Mitochondrial morphology was determined by MitoTracker Red CMXRos staining and confocal microscopy. Scale bars, 10 μm. **d ** The Pearson’s correlation coefficient (*R*^2^) of cofilin and MitoTracker colocalization was from 50 cells of five independent experiments. **e** Mitochondrial length was measured with ImageJ software. 50 cells of 5 independent experiments. **f**, **g** apoptosis was determined by Annexin V-FITC/PI staining and flow cytometry. **h** Western blot was performed to determine the expression of PARP, cleaved-PARP (CF), C-caspase 3 and cytochrome *c* in cytosolic fraction. Data represented as mean ± SD (*n* = 5, ***P < 0.001, Student’s two-tailed unpaired *t*-tests).

Human cofilin contains four cysteine residues including Cys39, Cys80, Cys139, and Cys147. Recent studies indicate that cofilin is oxidized at Cys39 and Cys80 under oxidative stress, leading to dephosphorylation of cofilin (Ser3) [37]. To explore the functional role of cofilin oxidation for its phosphorylation and mitochondrial translocation, we constructed the plasmids containing point mutations for Cys39 and Cys80 of cofilin. Mutants of G39-cofilin and G80-cofilin (Cys-39 or Cys-80 to Gly-39 or Gly-80) mimic oxidation of cofilin, and mutants of A39-cofilin and A80-cofilin (Cys-39 or Cys-80 to Ala-39 or Ala-80) mimic anti-oxidation of cofilin. Mutation of cofilin at Cys-39 or Cys-80 to Gly showed a conformational change of cofilin different from that of wild-type (WT)-cofilin during nonreducing SDS-PAGE, and G39-cofilin- and G80-cofilin-expressing cells treated with combination showed a similar conformational change of cofilin as that in these untreated cells. In contrast, A39-cofilin- and A80-cofilin-expressing cells treated without or with combination showed a similar conformational change of cofilin as that of WT-cofilin-expressing cells (Fig. 10a and Supplementary Fig. S10a). These findings indicate that Cys-39 and Cys-80 are the key sites for the oxidation of cofilin mediated by the combination.

We next employed cofilin cysteine mutants to analyze the functional role of cofilin oxidation for its Ser3 phosphorylation and mitochondrial translocation and the induction of cell death. Compared with WT-cofilin cells, minor phosphorylation of cofilin was observed in G39-cofilin- and G80-cofilin-expressing cells, and no phosphorylation of cofilin was observed in G39-cofilin- and G80-cofilin-expressing cells treated with the combination (Fig. 10b and Supplementary Fig. S10b). Furthermore, the mitochondrial translocation of cofilin was found in G39-cofilin- and G80-cofilin-expressing cells, and these events were enhanced by the combination treatment (Fig. 10b and Supplementary Fig. S10b). In contrast, the phosphorylation of cofilin was observed in A39-cofilin- and A80-cofilin-expressing cells treated without or with combination (Fig. 10b and Supplementary Fig. S10b), and A39-cofilin and A80-cofilin were not translocated to the mitochondria in cells treated without or with combination (Fig. 10b and Supplementary Fig. S10b). Finally, combination treatment led to increases in apoptosis in WT-cofilin expressing cells. However, the overexpression of G39-cofilin and G80-cofilin led to mild increases in mitochondrial fission and apoptosis even in the absence of combination treatment, and significantly enhanced combination-induced mitochondrial fission and apoptosis. In contrast, overexpression of A39-cofilin and A80-cofilin abrogated combination-induced mitochondrial fission and apoptosis (Fig. 10c–g and Supplementary Fig. S10c–g). Taken together, these findings indicate that the oxidation of cofilin at Cys39 and Cys80 residues is crucial for its dephosphorylation and mitochondrial translocation, mitochondrial fission, as well as apoptosis under combination treatment.

## Discussion

Preclinical studies have shown that the pharmacological inhibition of autophagy (with agents including chloroquine, hydroxychloroquine, metformin, etc) can enhance the cell-killing effect of cancer therapeutics, and a number of these agents are currently under investigation in clinical trials [20, 38, 39]. However, many of these autophagy modulators are relatively nonspecific and their cytotoxicity in noncancerous tissues is still a concern. Therefore, development of a new type of autophagy inhibitor for the treatment of cancer without these disadvantages has important clinical significance. In our previous study, we found that cepharanthine inhibited autophagy/mitophagy through blockage of autophagosome-lysosome fusion in human breast cancer cells. A mechanistic study revealed that downregulation of MYO1C mediated by cepharanthine inhibited autophagosome-lysosome fusion through inhibition of the F-actin network [22]. These findings suggest that cepharanthine could potentially be further developed as a novel autophagy/mitophagy inhibitor, and a combination of cepharanthine with classic chemotherapeutic agents (e.g., epirubicin) could represent a novel therapeutic strategy for treatment of breast cancer. Here we report that cepharanthine potentiates the efficacy of epirubicin in treatment of breast cancer. We also found that the synergistic effect of cepharanthine (2 μM) and epirubicin (0.1 μM) on apoptosis was more potent than that of CQ (20 μM) and epirubicin (0.1 μM). Because cepharanthine is an approved chemotherapeutic agent for treating a variety of diseases without significant side effect, it shows promise as a new chemosensitizer that may make up part of a potential combination therapy for treatment of breast cancer in clinical settings.

ROS play important roles in the regulation of diverse cellular signaling pathways, including those for survival and cell death [40]. Increasing evidence reveals that two major sources of ROS in cancer are mitochondria and nicotinamide adenine dinucleotide phosphate (NADPH) oxidases [41, 42]. Mitochondrial respiratory chain complexes are generally thought to be responsible for generating ROS, O^2•–^, and H_2_O_2_. Mitochondria are not only an important source of ROS, but also a target of ROS [43]. Mitochondria play a central role in regulating apoptosis through loss of mitochondrial membrane potential △*Ψ*m and release of cytochrome *c* (Cyto *c*) that results in the activation of caspases and subsequent cell death [44]. Mitochondrial superoxide production is a major cause of the cellular oxidative damage that could induce apoptosis. Several chemotherapeutic agents are proposed to generate mitochondrial superoxide through altered mitochondrial morphology that leads to cell death. For instance, cambogin, a naturally-occurring polycyclic polyprenylated acylphloroglucinol, displayed apoptosis-inducing effects in breast cancer cell lines. Mechanistical study showed that cambogin treatment increased mitochondrial superoxide, leading to the dissociation of Trx1 from ASK1, resulting in mitochondrial fission and apoptosis [45, 46]. In this study, we employed three antioxidants, for example, TBAP, a cell permeable SOD mimetic, catalase, and sodium formate, which primarily act on O^2•–^, H_2_O_2_, and OH·, respectively, to investigate the involvement of individual ROS species in cepharanthine/epirubicin combination-mediated apoptosis. Our results suggest that O_2_^•–^ plays an essential role in cepharanthine/epirubicin mediated apoptosis in TNBC cells. We have also provided direct evidence that mitochondrial superoxide (O_2_^•–^) may contribute to mitochondrial fission and apoptosis mediated by cepharanthine/epirubicin combination. First, combination of cepharanthine/epirubicin significantly increased the production of mitochondrial superoxide. Second, pretreatment with MitoQ (an antioxidant of mitochondrial superoxide) effectively attenuated the elevation of mitochondrial superoxide in cells treated with the combination. Third, pretreating with MitoQ significantly attenuated combination-induced mitochondrial fission, release of cytochrome *c*, as well as apoptosis. Together, these findings suggest that mitochondrial superoxide is primarily responsible for combination-induced mitochondrial fission and cell death in TNBC cells.

How ROS modify the structure and function of the signaling proteins, which lead to mitochondrial fission and apoptosis, is not well understood. One of mechanisms for posttranslational protein modification by ROS is a conformational change in the structure of protein kinases by formation of intramolecular disulfide bridges in cysteine linkages [47]. Cofilin is best known as an actin-depolymerizing factor, and it participates in regulation of mitochondrial dynamics and apoptosis [31]. Previous studies indicated that dephosphorylated cofilin translocates to the mitochondria, leading to induction of apoptosis [32]. Oxidation of cofilin by TnCl or H_2_O_2_ also causes cofilin to translocate to the mitochondria inducing apoptosis [9, 48, 49]. Mechanistically, treatment of cofilin with H_2_O_2_ leads to conformational change of cofilin by formation of an intramolecular disulfide bridge in cysteine linkages particularly in Cys39 and Cys80. However, the molecular mechanism by which mitochondrial superoxide induces oxidation of cofilin remains unclear. Here we provide evidence that combination of cepharanthine/epirubicin-induced mitochondrial superoxide results in oxidation of cofilin by formation of an intramolecular disulfide bridge. Interestingly, Cys39 and Cys80 are critical sites within the cofilin molecule with regard to its conformational change. Mutation of Cys39 and Cys80 to glycine, which mimic oxidation of cofilin, induced a similar conformational change of the cofilin molecule as observed after combination treatment, whereas mutation of Cys39 and Cys80 to alanine, which mimic anti-oxidation of cofilin, may inhibit disulfide bond formation without the induction of conformational changes. Oxidation of cofilin by TnCl or H_2_O_2_ leads to its dephosphorylation (Ser3) [12]. Consistent with these reports, our study showed that combination of cepharanthine/epirubicin-mediated oxidation of cofilin prevents its Ser3 phosphorylation. A likely possibility is that this combination, through a mechanism yet to be elucidated, inhibits the activities of LIM kinase. The findings that combination treatment efficiently reduced phosphorylation of LIMK and pretreating cells with MitoQ (a mitochondrial targeted antioxidant) effectively attenuated combination-inhibited phosphorylation of LIMK are consistent with this concept.

In summary, the present findings demonstrate that the inhibition of autophagy/mitophagy by cepharanthine enhanced epirubicin-induced apoptosis by triggering mitochondrial fission in TNBC cells. Mechanistically, combination of cepharanthine/epirubicin-induced mitochondrial superoxide represents a primary event resulting in oxidation of cofilin, leading to its Ser3 dephosphorylation and subsequent mitochondrial translocation, and culminates in mitochondrial fission and apoptosis (Supplementary Fig. S11). Most importantly, the present study provides a promising strategy of combining cepharanthine with epirubicin as a potential therapy for treatment of breast cancer.

## Supplementary information

Supplementary information
