# Supplementary material for: Cepharanthine sensitizes human triple negative breast cancer cells to chemotherapeutic agent epirubicin via inducing cofilin oxidation-mediated mitochondrial fission and apoptosis
Source: Acta Pharmacol Sin. 2021 Jul 22;43(1):177–93. doi: 10.1038/s41401-021-00715-3 (PMC8724299; doi:10.1038/s41401-021-00715-3)
Supplement: Supplementary file 1 — Supplementary information [file 41401_2021_715_MOESM1_ESM.docx]

**Cepharanthine sensitizes human triple negative breast cancer cells to chemotherapeutic agent epirubicin via inducing cofilin oxidation-mediated mitochondrial fission and apoptosis**

Supplementary


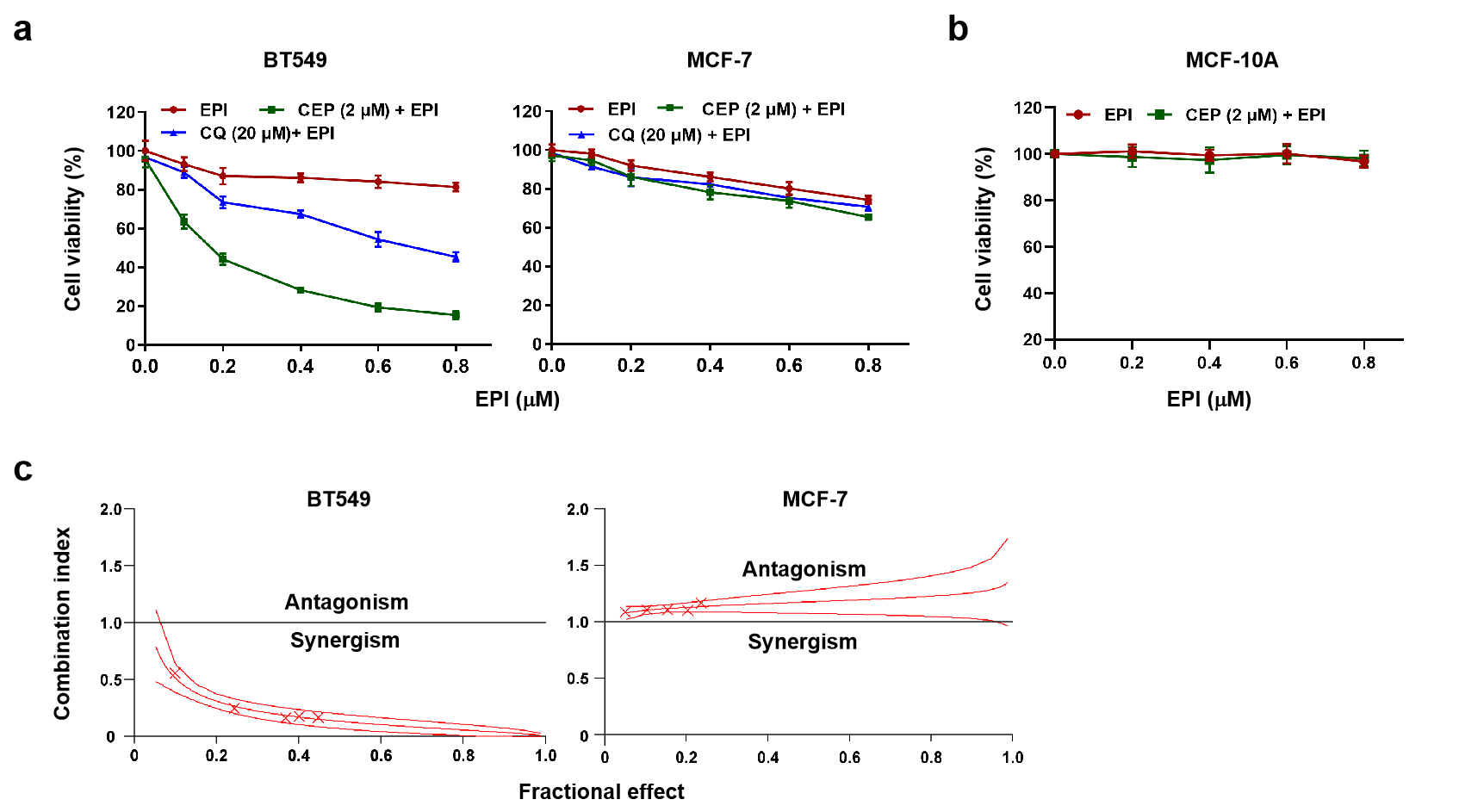


Supplementary Fig. 1 Synergistic effects of cepharanthine and epirubicin on cell proliferation in human breast cancer BT549, MCF-7 and normal breast epithelial MCF-10A cells. (a, b) BT549, MCF-7 and MCF-10A cells were treated with various concentrations of epirubicin (EPI) in the presence or absence of 2 μM cepharanthine (CEP) or 20 μM CQ for 48 h, and MTT assays were performed to assess cell proliferation. (c) The combination index (CI) values for each fraction affected were determined using commercially-available software (Calcusyn, Biosoft). CI values less than 1.0 correspond to synergistic interactions.


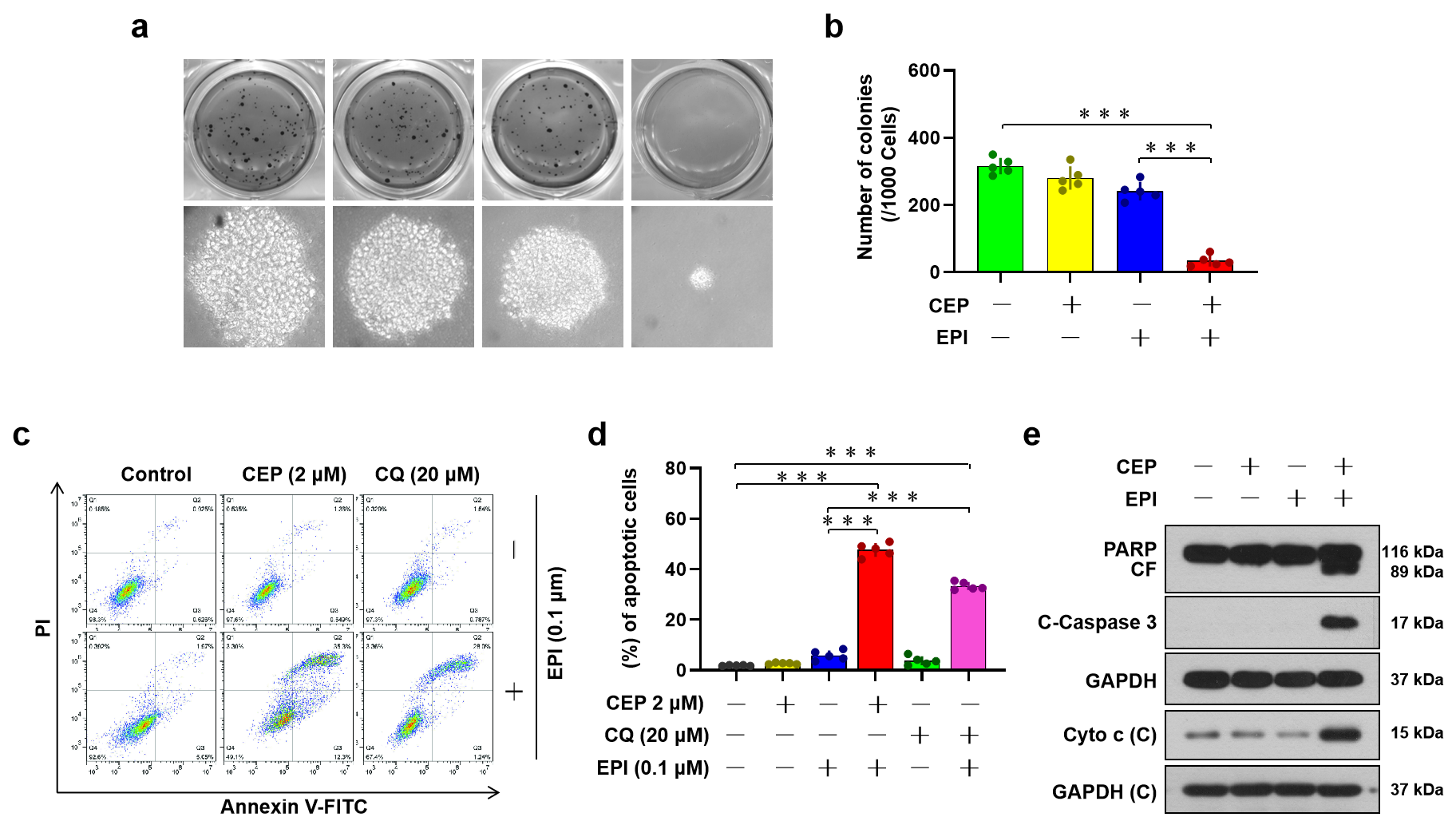


Supplementary Fig. 2 Combination of cepharanthine/epirubicin inhibits colony formation and induces apoptosis. (a, b) BT549 cells were cotreated with CEP and EPI, colony formation was detected using a soft agar assay. (c, d) Cells were treated with EPI in the presence or absence of CEP (2 μM) or 20 μM CQ for 48 h, Annexin V-FITC/PI staining and flow cytometry were employed to determine apoptosis. (e) Total cellular extract and cytosol fractions were prepared and subjected to western blot using antibodies against total PARP, cleaved-PARP (CF), cleaved caspase-3 (C-caspase 3), and cytochrome *c* (Cyto c). Data represented as mean ± SD (*n* = 5, ****P* < 0.001, Student’s two-tailed unpaired *t*-tests).


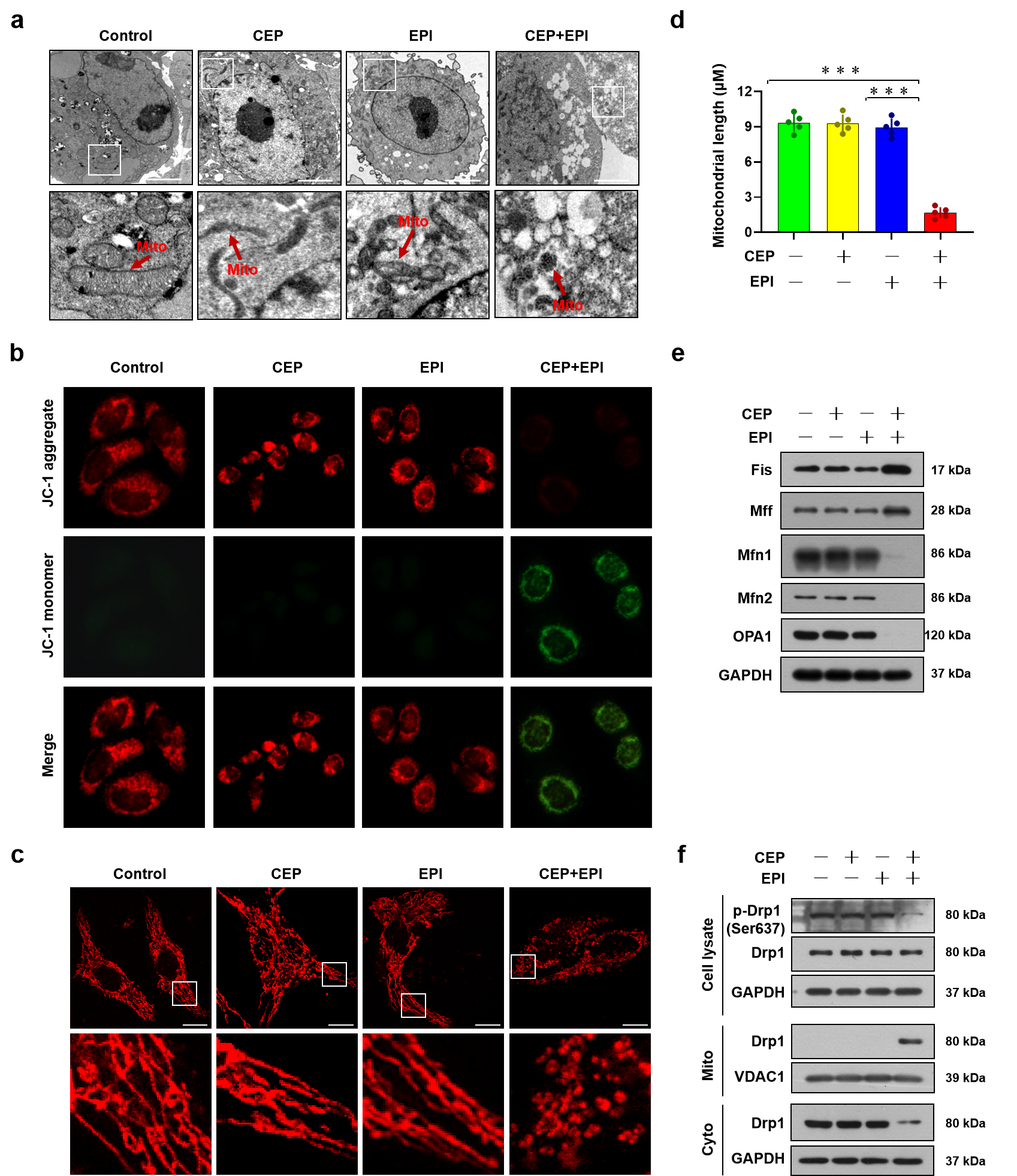


Supplementary Fig. 3 **Combination of** cepharanthine**/**epirubicin induces mitochondrial fission. BT549 cells were treated without or with CEP or EPI alone or combination of CEP**/**EPI for 48 h. (a) Representative images of transmission electronic microscopy. Scale bars, 2 μm. (b) The mitochondrial membrane potential (MMP) was detected by JC-1 staining. (c) Mitochondrial morphology was determined by MitoTracker Red CMXRos staining and confocal microscopy. Scale bars, 10 μm. (d) Mitochondrial length was measured with ImageJ software. (e) Western blot was performed to detect the expression of Fis1, MFF, Mfn1, Mfn2 and OPA1. (f) Whole cell lysates (Cell lysate), cytosolic (Cyto) and mitochondrial (Mito) fractions were prepared and subjected to Western blot by using antibody against Drp1 and phospho-Drp1. GAPDH and VDAC 1 were used as loading control. Data represented as mean ± SD (*n* = 5, ****P* < 0.001, Student’s two-tailed unpaired *t*-tests).


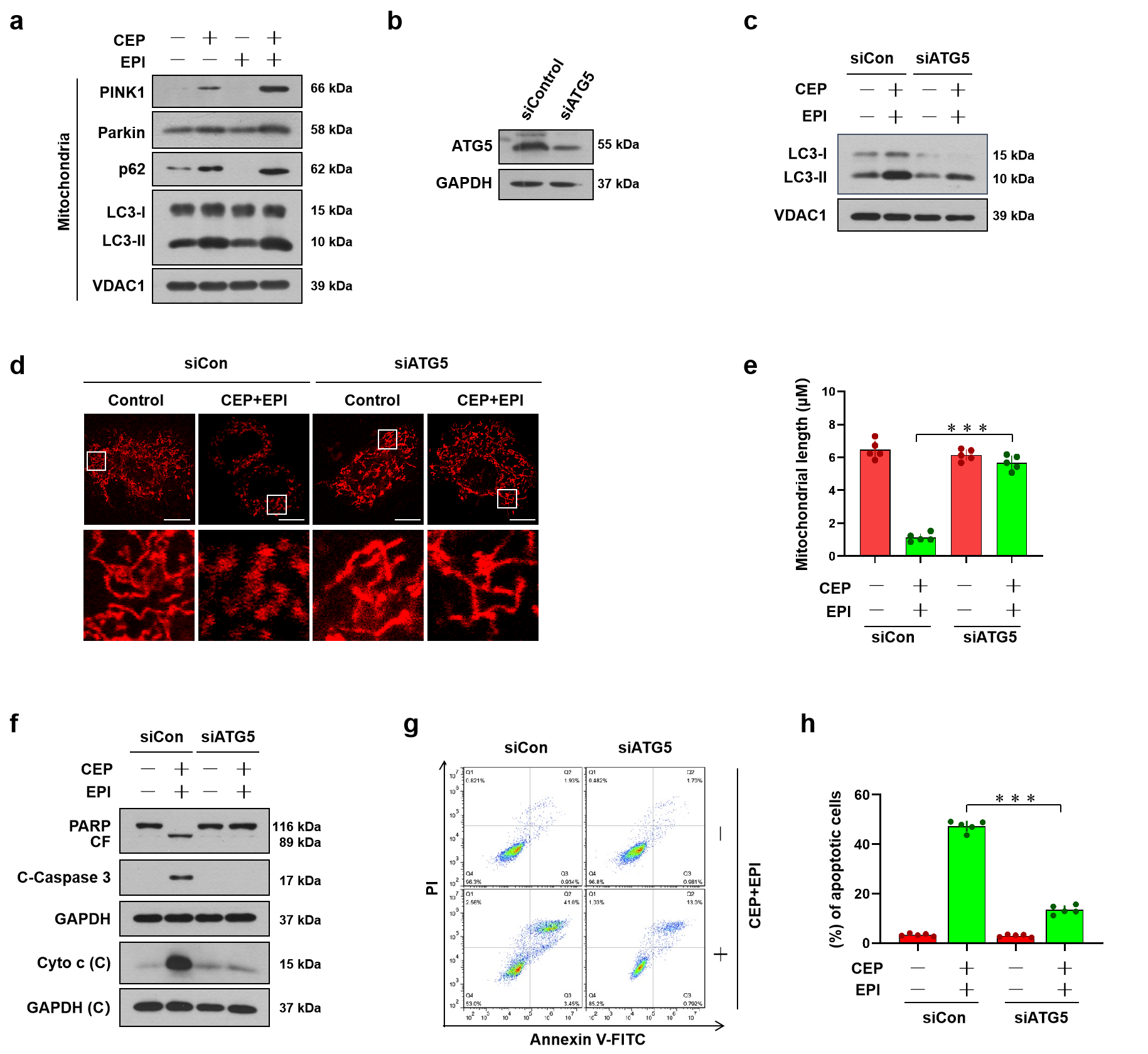


**Supplementary Fig. 4 Excessive accumulation of mitophagosomes contributes to apoptosis induced by cepharanthine/epirubicin combination.**

(a) BT549 cells were treated with cepharanthine in the presence or absence of epirubicin for 48 h, after which the mitochondrial fractions were prepared and subjected to Western blot analysis using antibodies against p62, LC3-I/LC3-II, PINK1 and Parkin. VDAC1 was used as loading control. (b) Cells were transfected with control siRNA (siControl) or siATG5, and Western blot analysis was used to determine the expression of ATG5. GAPDH was used as loading control. For c-h, cells stably expressing siControl or siATG5 were treated with cepharanthine in the presence or absence of epirubicin for 48 h. (c) The mitochondrial fractions were prepared and subjected to Western blot using antibodies against LC3-I/LC3-II. VDAC1 was used as loading control. (d) Mitochondrial morphology was determined by MitoTracker Red CMXRos staining and confocal microscopy. Scale bars, 10 μm. (e) Mitochondrial length was measured with ImageJ software. 50 cells of 5 independent experiments. (f) Western blot was performed to detect the expression of PARP, cleaved-PARP (CF), C-caspase 3 and cytochrome *c*. (g, h) Apoptosis was determined by Annexin V-FITC/PI staining and flow cytometry. Data represented as mean ± SD (*n* = 5, ****P* < 0.001, Student’s two-tailed unpaired *t*-tests).


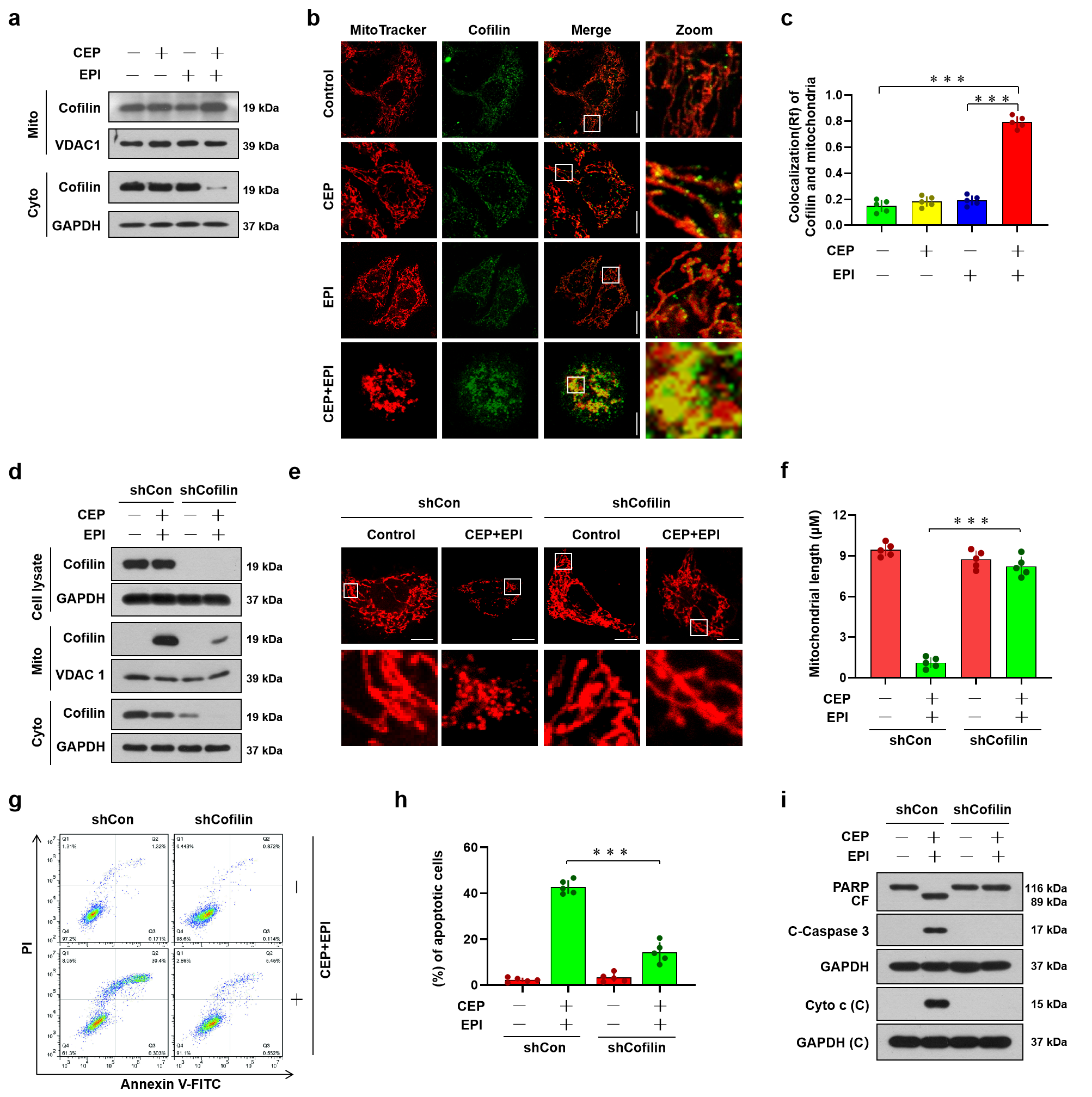


Supplementary Fig. 5 **Combination of** cepharanthine**/**epirubicin causes translocation of cofilin to the mitochondria. BT549 cells were treated without or with CEP or EPI alone or combination of CEP**/**EPI for 48 h. (a) Cytosolic and mitochondrial fractions were prepared and subjected to western blot using antibody against cofilin. (b) Representative images of confocal microscopy which showed the colocalization of cofilin (green) and MitoTracker (red). Scale bars, 10 μm. (c) The Pearson’s correlation coefficient (R^2^) of cofilin and MitoTracker colocalization was from 50 cells of five independent experiments. For d-i, BT549 cells stably expressing Non-Target shRNA (shCon) or cofilin shRNA (shCofilin) were treated with or without combination of CEP/EPI. (d) Whole cell lysates (Cell lysate), cytosolic (Cyto) and mitochondrial (Mito) fractions were prepared and subjected to western blot by using antibody against cofilin. (e) Mitochondrial morphology was determined by MitoTracker Red CMXRos staining and confocal microscopy. Scale bars, 10 μm. (f) Mitochondrial length was measured with ImageJ software. 50 cells of 5 independent experiments. (g, h) Apoptosis was determined by Annexin V-FITC/PI staining and flow cytometry. (i) Western blot was performed to detect the expression of PARP, cleaved-PARP (CF), cleaved caspase-3 (C-caspase 3), and cytochrome *c* (Cyto c). Data represented as mean ± SD (*n* = 5, ****P* < 0.001, Student’s two-tailed unpaired *t*-tests).


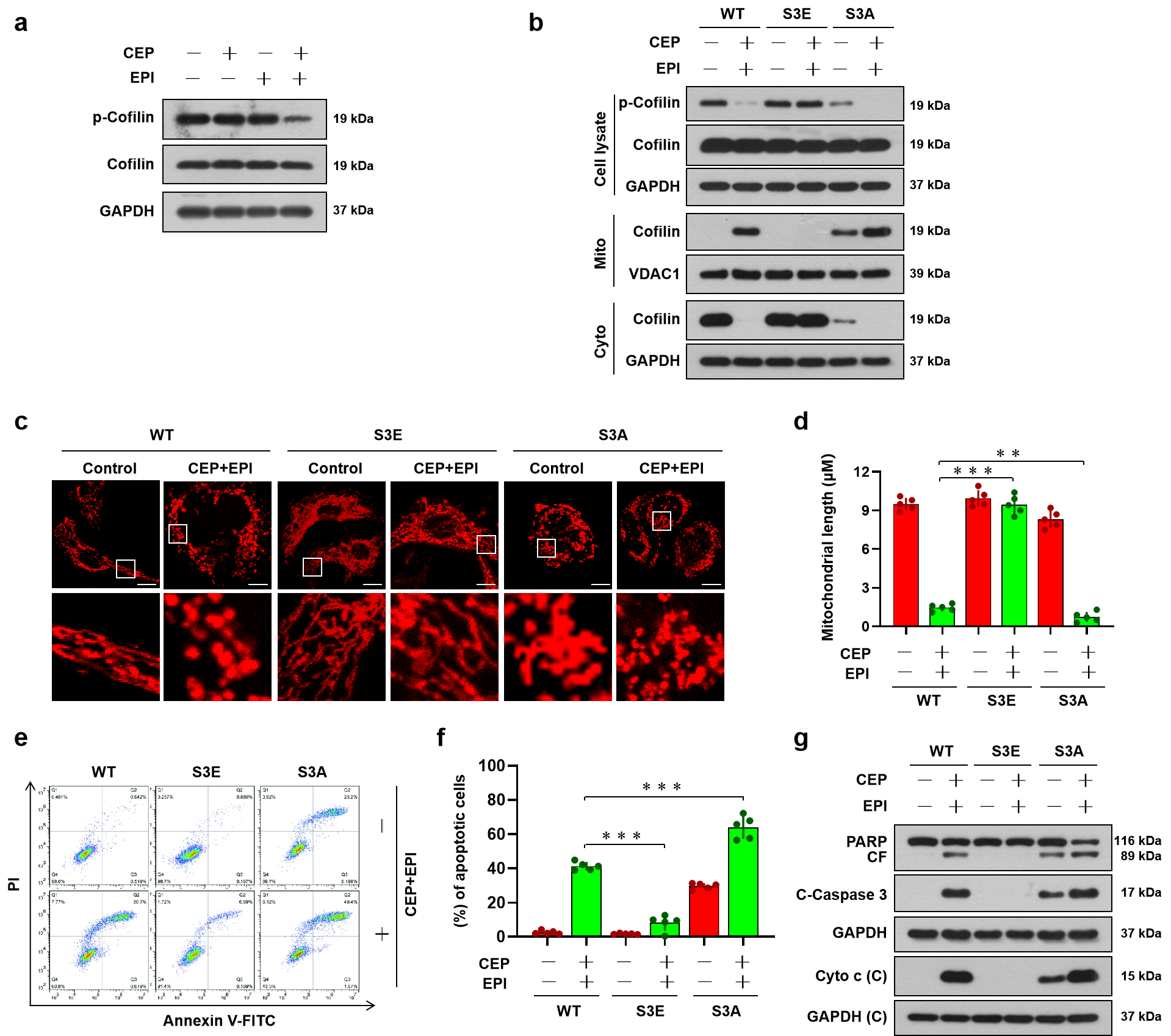


Supplementary Fig. 6 **Dephosphorylation of cofilin contributes to combination-mediated mitochondrial fission and apoptosis.** (a) BT549 cells were treated without or with CEP or EPI alone or combination of CEP**/**EPI for 48 h, after which western blot was employed to determine the levels of phospho-cofilin (p-Cofilin) and cofilin. For b-g, BT549 cells stably expressing Cofilin-WT or Cofilin-S3E or Cofilin-S3A were treated without or with combination of CEP/EPI. (b) Whole cell lysates (Cell lysate), cytosolic (Cyto) and mitochondrial (Mito) fractions were prepared and subjected to western blot by using antibody against cofilin and phospho-cofilin. (c) Mitochondrial morphology was determined by MitoTracker Red CMXRos staining and confocal microscopy. Scale bars, 10 μm. (d) Mitochondrial length was measured with ImageJ software. 50 cells of 5 independent experiments. (e, f) Apoptosis was determined by Annexin V-FITC/PI staining and flow cytometry. (g) Western blot was performed to detect the expression of PARP, cleaved-PARP (CF), cleaved caspase-3 (C-caspase 3), and cytochrome *c* (Cyto c). Data represented as mean ± SD (*n* = 5, ***P* < 0.01, ****P* < 0.001, Student’s two-tailed unpaired *t*-tests).


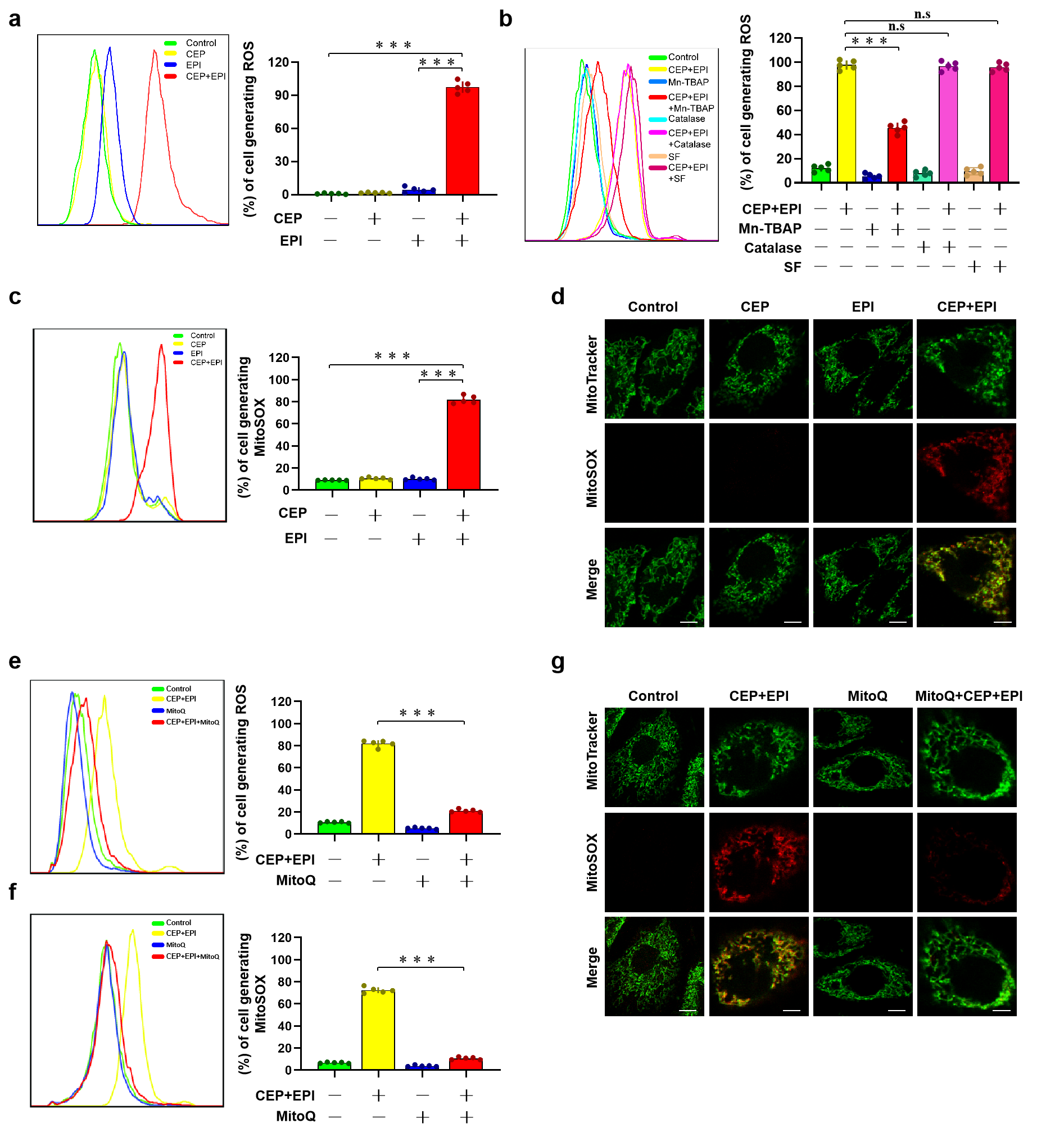


Supplementary Fig. 7 **Combination of** cepharanthine**/**epirubicin induces mitochondrial oxidative stress. (a) BT549 cells were treated without or with CEP or EPI alone or combination of CEP**/**EPI for 48 h, after which ROS production was determined by DCFHDA staining and flow cytometry. (b) Cells were pretreated with antioxidants including as TBAP (200 μM), catalase (5000 U/ml), and sodium formate (SF, 2 mM) for 1 h, followed by treatment with combination of CEP**/**EPI for 48 h, ROS production was determined by DCFHDA staining and flow cytometry. (c, d) Cells were treated in **a**, mitochondrial superoxide production was determined by the fluorescent probes MitoSOX™ staining and flow cytometry. Scale bars, 10 μm. (e, f) Cells were pretreated with mitoquinone (MitoQ), followed by treatment with combination of cepharanthine/epirubicin for 48 h, ROS and MitoSOX production was determined by DCFHDA or MitoSOX™ staining and flow cytometry. (g) The fluorescence intensity of mitochondrial superoxide as measured by MitoSOX and MitoTracker in cells treated without or with MitoQ or combination of cepharanthine/epirubicin. Scale bars, 10 μm. Data represented as mean ± SD (*n* = 5, ****P* < 0.001, ns not significant, Student’s two-tailed unpaired *t*-tests).


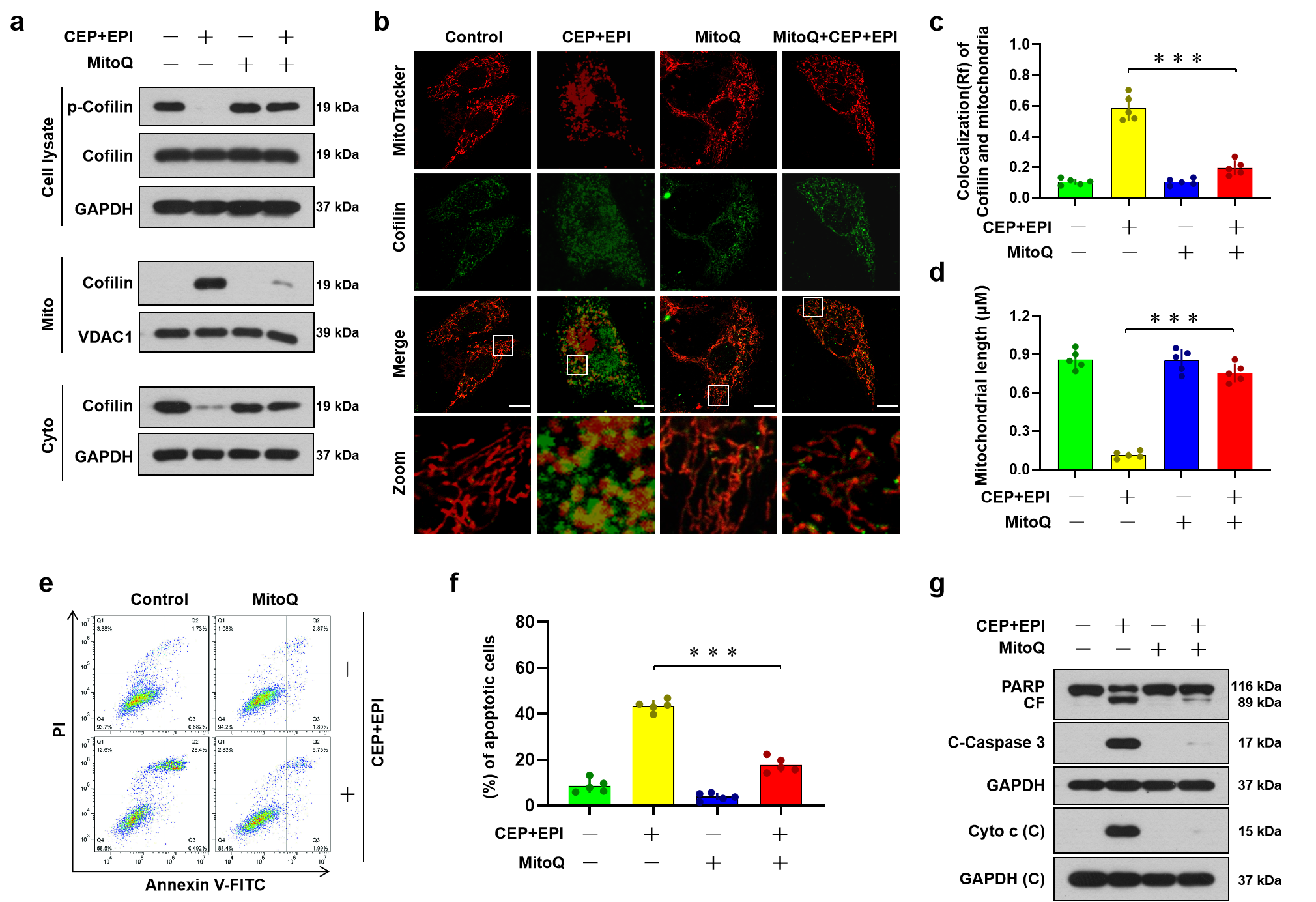


Supplementary Fig. 8 **Mitochondrial superoxide leads to dephosphorylation and mitochondrial translocation of cofilin.** BT549 cells were treated without or with combination of CEP**/**EPI in the presence or absence of MitoQ. (a) Whole cell lysates, cytosolic or mitochondrial fractions were prepared and subjected to western blot using antibodies against phospho-cofilin (Ser3) and cofilin. (b) Representative images of confocal microscopy which showed the colocalization of cofilin (green) and MitoTracker (red). Scale bars, 10 μm. (c) The Pearson’s correlation coefficient (R^2^) of cofilin and MitoTracker colocalization was from 50 cells of five independent experiments. (d) Mitochondrial length was measured with ImageJ software. 50 cells of 5 independent experiments. (e, f) Apoptosis was determined by Annexin V-FITC/PI staining and flow cytometry. (g) Western blot was performed to determine the expression of PARP, cleaved-PARP (CF), cleaved caspase-3 (C-caspase-3), and cytochrome *c* (Cyto c). Data represented as mean ± SD (*n* = 5, ****P* < 0.001, Student’s two-tailed unpaired *t*-tests).


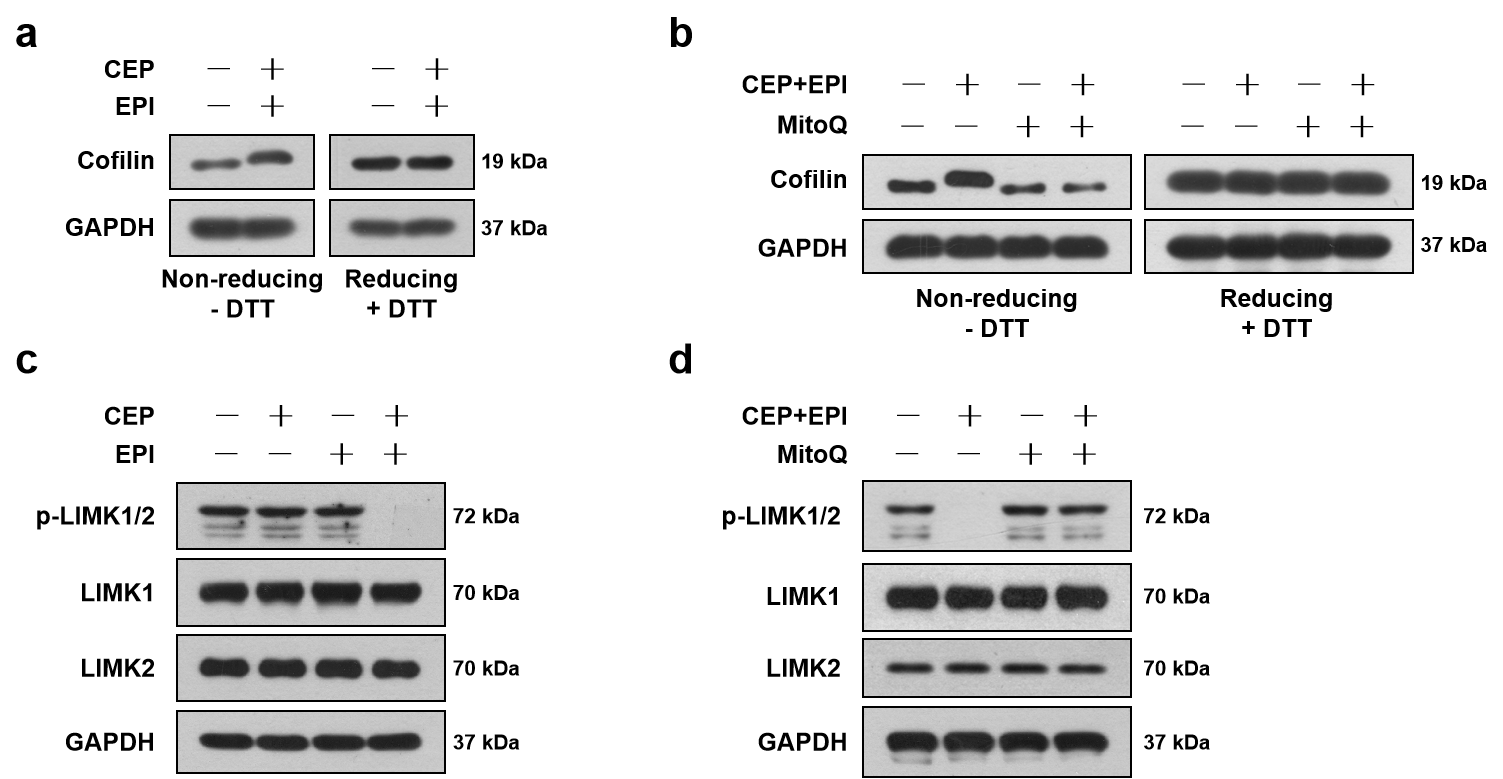


Supplementary Fig. 9 Combination-mediated mitochondrial superoxide leads to oxidation of cofilin prevents its phosphorylation (Ser3) by inactivation of LIM kinase. (a) BT549 cells were treated without or with combination of CEP**/**EPI, the expression of cofilin was analyzed either by nonreducing SDS-PAGE (left, DTT) or by reducing SDS-PAGE (right, +DTT). (b) Cells were treated without or with combination of CEP**/**EPI in the presence or absence of MitoQ, the expression of cofilin was analyzed either by non reducing SDS-PAGE (left, DTT) or by reducing SDS-PAGE (right, +DTT). (c) BT549 cells were treated without or with CEP or EPI alone or combination of CEP**/**EPI for 48 h, after which western blot was performed to determine the expression of phospho-LIMK1/2, LIMK1 and LIMK2. (d) Cells were treated without or with combination of CEP**/**EPI in the presence or absence of MitoQ, the expression of phospho-LIMK1/2, LIMK1 and LIMK2 was analyzed by western blot.


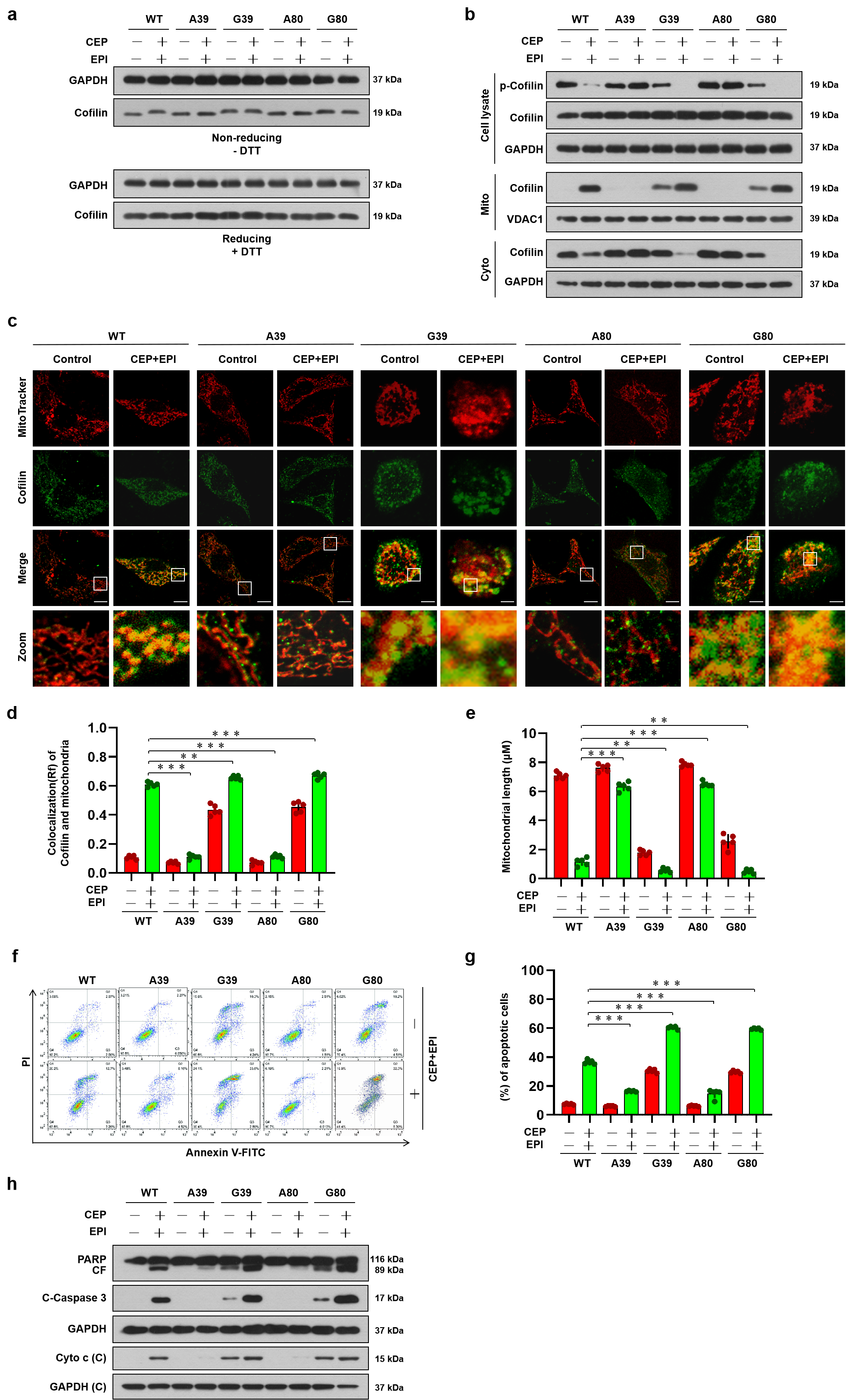


Supplementary Fig. 10 **Cys39 and Cys80 are key sites for combination-mediated dephosphorylation and mitochondrial translocation of cofilin.** BT549 cells expressing either FLAG-tagged WT-cofilin or cysteine-to-glycine mutants of cofilin (C39G and C80G) or cysteine-to-Ala (C39A and C80A) were treated without or with combination of CEP**/**EPI for 48 h. (a) The expression of cofilin was analyzed either by nonreducing SDS-PAGE (left, DTT) or by reducing SDS-PAGE (right, +DTT). (b) Whole cell lysates, cytosolic and mitochondrial fractions were prepared and subjected to western blot by using antibodies against phospho-cofilin and cofilin. (c) Mitochondrial morphology was determined by MitoTracker Red CMXRos staining and confocal microscopy. Scale bars, 10 μm. (d) Mitochondrial length was measured with ImageJ software. 50 cells of 5 independent experiments. (e) The Pearson’s correlation coefficient (R^2^) of cofilin and MitoTracker colocalization was from 50 cells of five independent experiments. (f, g) Apoptosis was determined by Annexin V-FITC/PI staining and flow cytometry. h Western blot was performed to determine the expression of PARP, cleaved-PARP (CF), cleaved caspase-3 (C-caspase-3), and cytochrome *c* (Cyto c). Data represented as mean ± SD (*n* = 5, ***P* < 0.01, ****P* < 0.001, Student’s two-tailed unpaired *t*-tests).


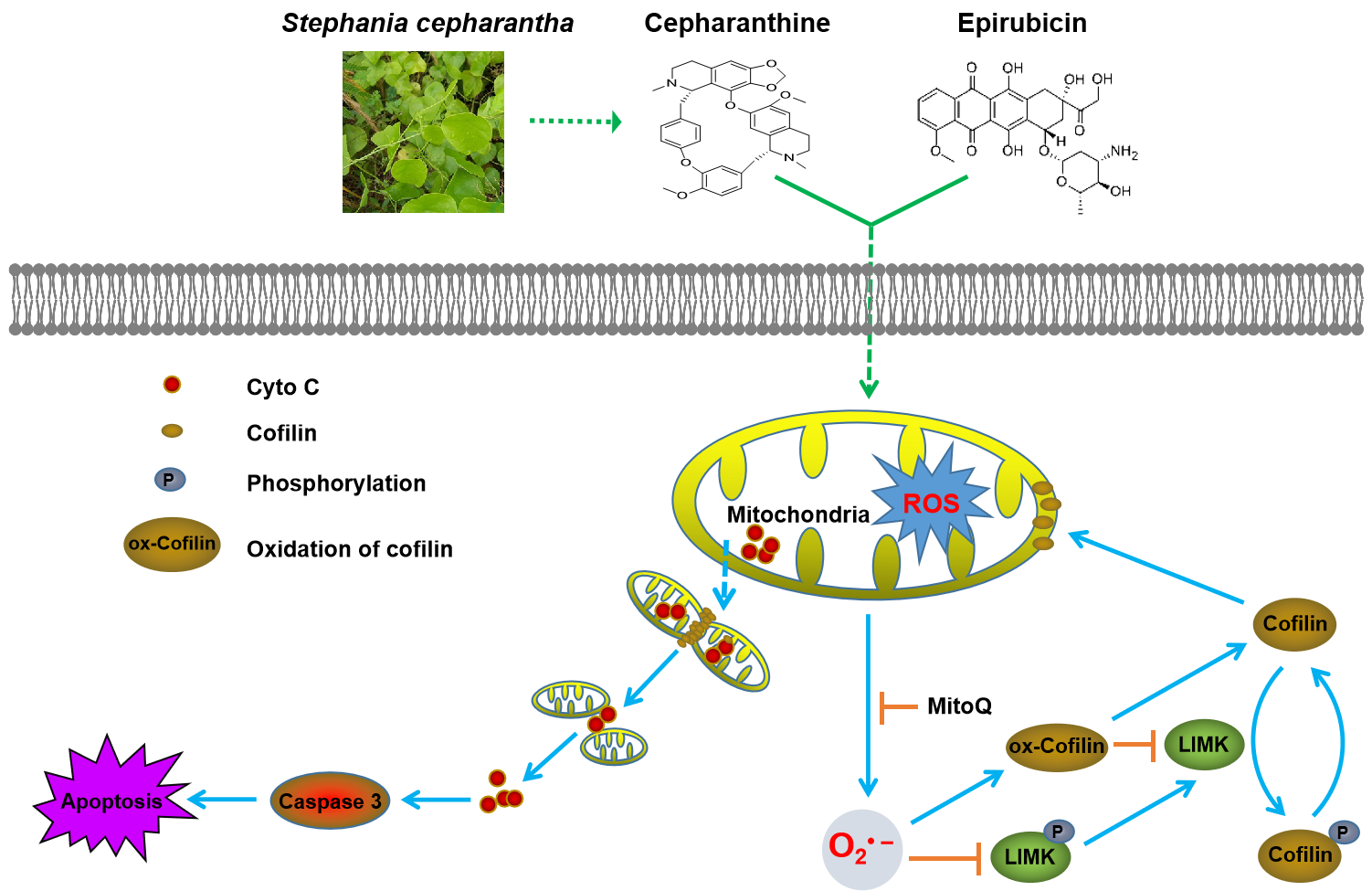


Supplementary **Fig. 11 A proposed model of oxidation of cofilin-mediated mitochondrial fission and apoptosis induced by combination of cepharanthine and epirubicin.** The combination of cepharanthine/epirubicin induces mitochondrial superoxide that represents a primary event resulting in oxidation of cofilin, leading to dephosphorylation and mitochondrial translocation of cofilin, and culminating in mitochondrial fission and apoptosis.
